# Genetic load has potential in large populations but is realized in small inbred populations

**DOI:** 10.1111/eva.13216

**Published:** 2021-04-10

**Authors:** Samarth Mathur, J. Andrew DeWoody

**Affiliations:** ^1^ Department of Biological Sciences Purdue University West Lafayette Indiana USA; ^2^ Department of Forestry and Natural Resources Purdue University West Lafayette Indiana USA; ^3^ Present address: Department of Evolution, Ecology and Organismal Biology The Ohio State University Columbus Ohio USA

**Keywords:** genetic erosion, genic variation, Montezuma quail, mutation load, variant effects

## Abstract

Populations with higher genetic diversity and larger effective sizes have greater evolutionary capacity (i.e., adaptive potential) to respond to ecological stressors. We are interested in how the variation captured in protein‐coding genes fluctuates relative to overall genomic diversity and whether smaller populations suffer greater costs due to their genetic load of deleterious mutations compared with larger populations. We analyzed individual whole‐genome sequences (*N* = 74) from three different populations of Montezuma quail (*Cyrtonyx montezumae*), a small ground‐dwelling bird that is sustainably harvested in some portions of its range but is of conservation concern elsewhere. Our historical demographic results indicate that Montezuma quail populations in the United States exhibit low levels of genomic diversity due in large part to long‐term declines in effective population sizes over nearly a million years. The smaller and more isolated Texas population is significantly more inbred than the large Arizona and the intermediate‐sized New Mexico populations we surveyed. The Texas gene pool has a significantly smaller proportion of strongly deleterious variants segregating in the population compared with the larger Arizona gene pool. Our results demonstrate that even in small populations, highly deleterious mutations are effectively purged and/or lost due to drift. However, we find that in small populations the realized genetic load is elevated because of inbreeding coupled with a higher frequency of slightly deleterious mutations that are manifested in homozygotes. Overall, our study illustrates how population genomics can be used to proactively assess both neutral and functional aspects of contemporary genetic diversity in a conservation framework while simultaneously considering deeper demographic histories.

## 1 INTRODUCTION

Many species and populations are declining at an alarming rate (Barnosky et al., 2011; Ceballos et al., 2015; Dirzo et al., 2014), mainly driven by human‐mediated habitat loss and climate change (Loarie et al., 2009). Active prevention of population declines and extirpations is a priority for conservation (Cardinale et al., 2012; Thompson et al., 2017) because reduction in population size is often followed by reduction in genetic diversity (Allendorf et al., 2013; Soulé, 1985). The loss of genetic diversity has negative consequences on the future persistence of a species as it impedes its ability to adapt to environmental change (Bijlsma & Loeschcke, 2005; Bürger & Lynch, 1995; Reed & Frankham, 2003). Smaller and/or isolated populations exhibit a more rapid loss of within‐population genetic variation as compared to their larger counterparts (Willi et al., 2006). The combined effects of drift, inbreeding, weak selection, and lack of gene flow in small, isolated populations may lead to “genetic erosion” (Bijlsma & Loeschcke, 2012). Genetic erosion can impede future adaptive potential in small inbred populations (Keller, 2002), reduce the mean fitness of a population, and increase extinction risks (Bijlsma & Loeschcke, 2012; Leroy et al., 2018).

In theory, mean fitness is expected to progressively decrease in small isolated populations because of the accumulation of deleterious mutations that are ineffectively purged by selection. In large populations and/or when selection intensity is very strong (i.e., when *N*_e_(*s*) > 1, where *N*_e_ is effective population size and *s* is the selection coefficient), natural selection is an effective determinant of allelic fate (Kimura & Ohta, 1969). However, in small populations and/or when selection is weak (e.g., on small‐effect mutant alleles), genetic drift is more pronounced and allelic fate is more stochastic (Lynch et al., 1995). Thus, highly deleterious mutations are more likely to be purged by selection than to drift to high frequencies, whereas slightly deleterious mutations can actually increase in frequency in small populations (Hedrick & Garcia‐Dorado, 2016). Most of the genes underlying adaptation represent complex polygenic traits, and most genetic load is probably due to small‐effect (i.e., only slightly deleterious) alleles (Charlesworth & Charlesworth, 1987), which indicates that genetic erosion can reduce mean fitness of small populations if small‐effect recessive deleterious alleles rise in frequency due to drift (Charlesworth et al., 1993; Lynch, 2007).

In practice, empirical evidence for the purging of deleterious mutations is mostly experimental and there is far less evidence from natural populations, especially with respect to genomic sequence data (Bersabé & García‐Dorado, 2013; Bijlsma et al., 1999; Crnokrak & Barrett, 2002; Grossen et al., 2020; Rettelbach et al., 2019). Economic and technical breakthroughs in whole‐genome resequencing now make such assessments in wild populations far more tractable. Beyond the basic evolutionary interest in allelic fates, the genetic erosion of adaptive potential is increasingly recognized as a major threat to modern conservation efforts (Holderegger et al., 2019; Ralls et al., 2018).

Much of the vertebrate genome is thought to evolve in a neutral or nearly neutral fashion (Ohta, 1992) and is shaped by genome‐wide processes such as inbreeding, migration, and demographic stochasticity (Pool & Nielsen, 2007). For example, contemporary genomic patterns of neutral diversity may be affected by the recent lack of gene flow due to anthropogenic habitat fragmentation and historic demographic responses to glaciations (Nadachowska‐Brzyska et al., 2015). Beyond neutrality, variants in genic regions often underlie evolutionary adaptations subject to natural selection, and the mode and strength of selection largely determine the phenotypic response (Ellegren & Sheldon, 2008). Hence, explicitly comparing whole genomes with defined genic regions should help with identifying the major contributors to overall genomic architecture and also gauge the adaptive potential of populations. In this study, we use whole‐genome sequences to quantify genic and whole‐genome variation from different‐sized populations of Montezuma quail (*Cyrtonyx montezumae*), and then estimate the degree of genetic erosion and its impact on adaptive potential by investigating the genetic load via biochemical predictions as inferred from coding regions throughout the genome.

**FIGURE 1:**
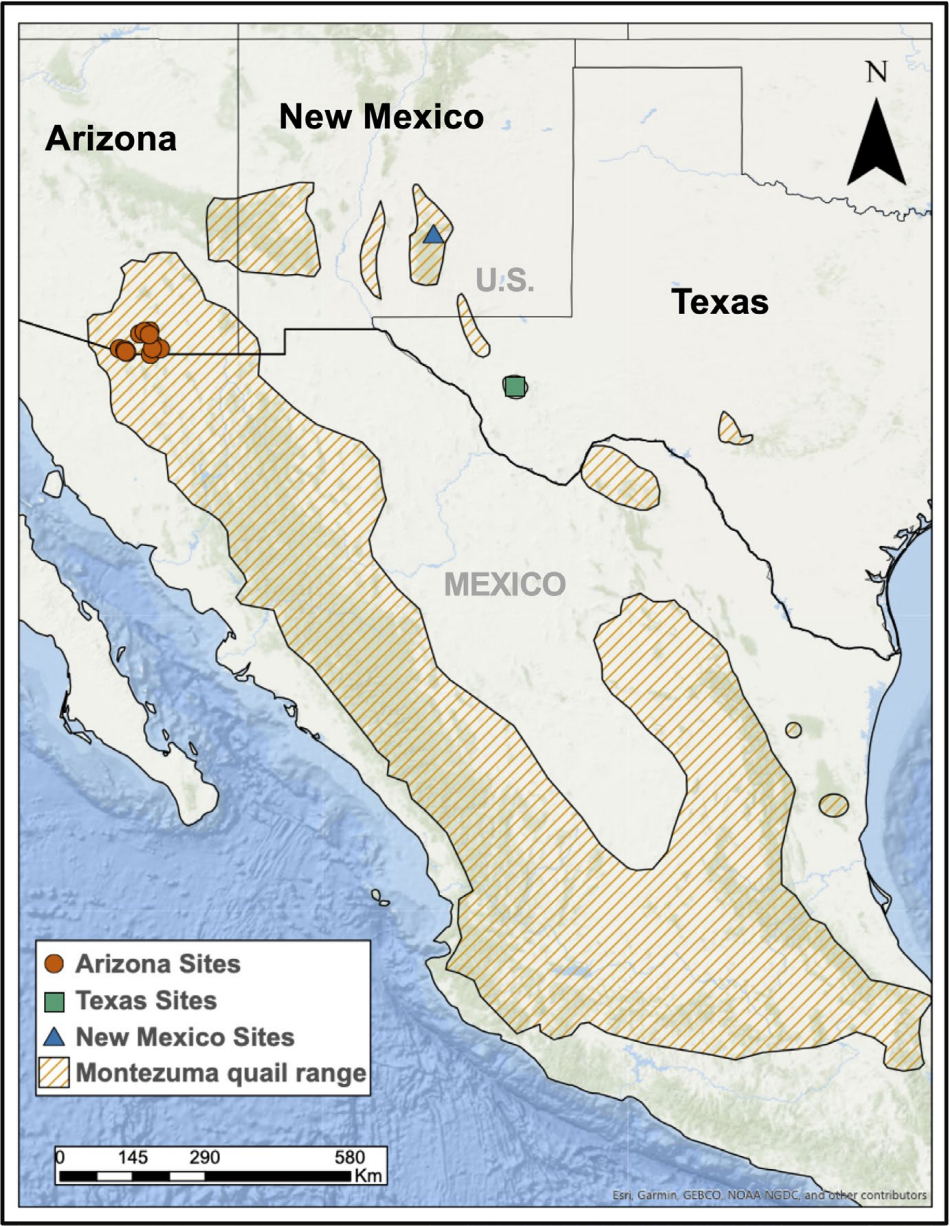
Montezuma quail species range and sampling sites (from Mathur et al., 2019). Samples were collected from the larger and most contiguous Arizona sites (*N* = 60), from an intermediate‐sized population in New Mexico (*N* = 13), and from a relatively isolated and small population in Texas (*N* = 15)

The Montezuma quail is a small game bird that is hunted in portions of Mexico, New Mexico, and Arizona but of conservation concern in Texas (Figure 1). It is one of the least‐studied avian species in North America (Gonzalez Gonzalez, Harveson, & Luna, 2017) due to its cryptic nature and difficulties associated with live trapping and monitoring (Hernandez et al., 2006). Montezuma quail are currently experiencing species‐wide declines within the United States (Harveson et al., 2007), and Texas populations are listed as Vulnerable by Texas Parks and Wildlife Department (TPWD) with no open hunting season due to growing concerns about extirpations (Harveson, 2009). Unlike other North American quails, Montezuma quail are diet (Albers & Gehlbach, 1990) and habitat specialists (Brown, 1979) that heavily rely on grass cover for predator evasion (Bristow & Ockenfels, 2004). Their demography is strongly impacted by seasonal rainfall (Chavarria et al., 2012) and adequate grass cover (Brown, 1979), making habitat degradation and fragmentation major threats to Montezuma quail survival (Luna et al., 2017). Populations in Arizona are more genetically diverse than those from Texas or New Mexico (Mathur et al., 2019) and are expected to be the least impacted by genetic erosion due to larger sizes and more contiguous habitat (Figure 1). In contrast, the Texas population is expected to have the highest signature of genetic erosion due to a restricted geographic range and associated demographic isolation (Mathur et al., 2019).

Herein, we report the data from whole‐genome sequencing (WGS) of 90 Montezuma quail from Arizona, New Mexico, and Texas. We used these WGS data to quantify the levels of overall genomic diversity, genic variation, differentiation, individual inbreeding, and the inferred genetic load in each population. We do so in a conservation context by comparing populations of different sizes. Our results indicate that Montezuma quail effective population sizes have decreased over much of the last million years, and their similar trajectories over time indicate that now‐disjunct populations in the United States were long connected demographically. Furthermore, we find that the small Texas population is isolated and genetically depauperate, and that its genetic load is mostly due to small‐impact deleterious mutations. Because inbreeding is also more pronounced in the small Texas population compared with the larger populations, these deleterious mutations are more likely to occur in homozygotes and thus contribute to a decline in fitness.

## 2 MATERIALS AND METHODS

### 2.1 Samples, DNA extraction, and sequencing

Montezuma quail samples were opportunistically collected (i.e., either hunter‐harvested wing tissues or roadkill carcasses) from three representative geographic populations in the United States: Arizona (AZ), New Mexico (NM), and Texas (TX) as described earlier (Mathur et al., 2019; Figure 1). Based on the size of their geographic range in each state, on assessments by each state game agency, on eBird sightings, and on previous genetic analyses, we explicitly assume that AZ samples come from a large population, NM from a medium‐sized population, and TX from a small population relative to each other (Mathur et al., 2019). Arizona samples were acquired from hunter‐harvested wings initially collected by Randel et al. (2019). New Mexico samples were acquired as voucher specimens by R. Luna, whereas Texas samples were collected as roadkill carcasses by L. Harveson. Sample handling and DNA extraction protocols are described in Mathur et al. (2019).

We sequenced whole genomes of 90 Montezuma quail samples (AZ = 60, TX = 17, and NM = 13) by creating individually barcoded dual‐index libraries using Illumina^®^ Nextera™ reagents following the manufacturer's protocol. The libraries were sequenced in 8 lanes of paired‐end 150 bp reads (2 × 150 bp) on one S4 flow cell using Illumina^®^ NovaSeq™ 6000 sequencing system in Purdue University's Genomics Core Facility. The estimated genome size of Montezuma quail is 1.03 Gb (Mathur et al., 2019), and we removed any sample if they failed to generate more than 8 million reads (i.e., less than 1× mean read depth).

### 2.2 Sequencing filtering, alignment, and read preprocessing

We used FastQC v0.11.7 (Andrews, 2010) to quality check our raw reads and removed adapter sequences from trailing and leading edges of each read using Trimmomatic v.036 (Bolger et al., 2014). We also used Trimmomatic to remove low‐quality sequences (Phred <20) and any read smaller than 30 bp after clipping and quality filtering, prior to any further downstream analysis.

The filtered reads were mapped to a Montezuma quail draft genome assembly (Mathur et al., 2019) with BWA v.0.7.17 (Li & Durbin, 2009) using the *mem* algorithm. Our final dataset contained 74 individuals (AZ = 52, TX = 15, and NM = 7). We used the Genome Analysis ToolKit (GATK) “Best Practice Workflow” (Van der Auwera et al., 2013) to preprocess our mapped reads. We first sorted the reads by their coordinates and marked duplicates using Picard Tools (http://picard.sourceforge.net). We then used GATK v3.6.0 (McKenna et al., 2010) to realign our reads around indels to minimize misaligning with mismatches. We identified the regions to be realigned using RealignerTargetCreator and aligned BAM files using IndelRealigner. The base quality score was recalibrated for all the reads using known variant sites discovered from high coverage genome reads (Mathur et al., 2019) using BaseRecalibrator. We finally used these filtered–realigned–recalibrated reads to get coverage statistics using SAMtools depth (Li et al., 2009) and for further downstream analyses.

In cases where we needed to polarize genomic variants as ancestral or derived (i.e., for selection scan and population trend analyses; see below), we used the high‐quality and contiguous chicken genome (*Gallus gallus* GRCg6a) as reference. Both Galliformes, Montezuma quail, belong to the New World quail family Odontophoridae that diverged from junglefowl (*Gallus* spp.; family: Phasianidae) approximately 30–40 million years ago (Cox et al., 2007; Hosner et al., 2015). Read mapping and preprocessing steps were the same as above.

### 2.3 Mitogenome assembly and diversity

We mapped genomic reads to the previously published Montezuma quail mitogenome (Mathur et al., 2019) and extracted the uniquely mapped reads (mito‐reads) using BBMap v37.93 (Bushnell, 2014). Since nuclear copies of mitochondrial DNA (NUMTs) exist in nearly all eukaryotic genomes (Bensasson et al., 2001; Lopez et al., 1994), we tried to first identify the NUMTs in the nuclear genome assembly of the Montezuma quail. We used a BLAST‐based approach to query the Montezuma quail reference mitogenome against a custom blast database of Montezuma quail nuclear genome scaffolds. We extracted the NUMT sequences from genome assembly as FASTA files using faSomeRecords (Kent et al., 2002). Any mito‐read that also uniquely matched to the NUMT fasta sequences were removed using BBMap. This helped ensure that final mito‐specific reads we retained belonged to the mitogenome and not NUMTs. We used SAMtools mpileup to align mito‐specific reads to the reference mitogenome and used bcftools (Li et al., 2009) to call variants. We filtered the variants with a minimum base depth of 10 using vcflib (Garrison, 2012) and used bcftools consensus to create consensus mitogenomes for every individual. To avoid mismapping and errors introduced at the artificial ends created in the linearized mitogenome, we trimmed 40 bp from either end of the mitochondrial sequence prior to analysis.

All mitogenomes were aligned as multiple sequence alignment using Clustal W v.2.1 (Thompson et al., 1994) using default parameters. We calculated mitochondrial nucleotide diversity indices and haplotype statistics using Arlequin v3.5 (Excoffier & Lischer, 2010). We accounted for unequal sample sizes for each population by randomly subsampling mitochondrial genomes from each population (*N* = 7) and recalculated nucleotide diversity indices using 100 independent permutations.

### 2.4 Genotype likelihood estimation, subsampling, and genotype calling

For the nuclear reads, we used the SAMtools model in ANGSD v0.929 (Korneliussen et al., 2014) to estimate genotype likelihoods (GLs) and call single nucleotide polymorphisms (SNPs). We filtered BAM files to only include unique reads with a minimum mapping quality of 20. We excluded bases with a base quality score <20 and only retained only proper pairs. Major and minor allele was inferred from the GL, and triallelic sites were removed. Per‐site allele frequencies (AFs) were estimated using a combination of estimators, that is, first estimating AF from GL assuming both major and minor alleles are known and then re‐estimating AF by summing over the three possible minor alleles weighted by their probabilities. We used a *p*‐value cutoff of 10^−6^ to call a site polymorphic and a minimum minor allele frequency (MAF) of 0.05. We also used a maximum depth threshold of 500 to avoid calling SNPs from repetitive regions (Clucas et al., 2019). Deviations from the Hardy–Weinberg equilibrium were tested, and sites with *p*‐value <0.01 were filtered out to remove potential paralogous sequences with an excess of heterozygotes due to erroneous mapping (Meisner & Albrechtsen, 2019).

When estimating GL across all samples (*N* = 74), we used a threshold of minimum 60 individuals to ensure genotypic information is captured in at least 80% of all samples and to avoid retaining segregating sites from only the Arizona population (*N* = 52) (“population dataset”). To avoid biases introduced due to uneven sample sizes, we re‐estimated GL and discovered SNPs from an equal subset of Arizona and Texas samples (*N* = 21; AZ = 7, TX = 7, and NM = 7). For our subsamples, we chose samples with the highest depth and breadth of coverage to maximize the genomic spread of our variants (“genomic dataset”). For the subset, we used a minimum individual threshold of 15 and maximum depth threshold of 100.

In the end, we analyzed our GL data in two ways: (a) retaining maximum individual information at the cost of markers per individual (“population dataset”) and (b) retaining maximum genomic information on each population at the cost of individuals analyzed per population (“genomic dataset”). The population dataset was used for the estimation of inbreeding and genetic structure, both of which can be inferred from a smaller set of widespread markers from more individuals (McLennan et al., 2019), whereas the genomic dataset with higher SNP density was used to estimate genome‐wide diversity and for detecting signatures of selection (Benjelloun et al., 2019).

### 2.5 Relatedness, inbreeding coefficient, and population structure estimation

Assumptions of many population genetic estimators are violated if family members and closely related individuals are analyzed simultaneously. Related individuals among a sample set should thus be identified and removed prior to population structure analysis (Meisner & Albrechtsen, 2018, 2019). We estimated relatedness among our samples using IBSrelate (Waples et al., 2019). IBSrelate uses GL estimates to categorize a pair of individuals as either parent–offspring, full siblings, half‐siblings, first cousins, or unrelated based on whether the pair share the same genotype or exhibit dissimilar genotypes at a particular site (Manichaikul et al., 2010). We compared all individual pairs (total of 2701 comparisons) and removed any pairwise comparison from relatedness estimates if the number of sites compared was <100,000.

We estimated individual inbreeding coefficients (*F*) using PCAngsd v.0.982 (Meisner & Albrechtsen, 2018) from inferred GL. This allows *F*‐values at a site to vary between −1 and 1, where a negative value indicates an excess of heterozygotes and a positive value indicates an excess of homozygotes at a site. Since inbred individuals would have an excess of homozygous sites, they should have an overall *F* > 0. We used extremely low tolerance values (1 × 10^−9^) and 5000 maximum iterations for estimation to assure a stricter stopping criterion and avoid convergence at a local minimum (Figure S11).

To identify genetic structure in our Montezuma quail samples, we used two approaches: First, we used PCAngsd to calculate a covariance matrix and performed individual level PCA using princomp function in R (Team, 2013); second, we used NGSAdmix (Skotte et al., 2013) to estimate individual admixture proportions. For PCAngsd, we used a minimum tolerance value for population AF estimation of 1e‐9, a tolerance threshold for updating individual AF of 1e‐9 for 1000 iterations. For NGSAdmix, we ran 10 independent runs for each *K* from 1 to 10 with minimum MAF 0.05, 1e‐9 tolerance for convergence, 1e‐9 tolerance for log‐likelihood difference in 50 iterations, and maximum 50,000 iterations. The most likely number of subpopulations was determined based on first‐ and second‐order rate of change in the likelihood distribution from the 10 runs (Evanno et al., 2005).

### 2.6 Nucleotide diversity, heterozygosity, and contemporary effective population size estimation

For nucleotide diversity estimates, we only used the genomic dataset to avoid biases in estimating site frequency spectrum (SFS) due to uneven sample sizes and heavy data pruning, which was the case for our population dataset. We used ANGSD to generate a folded SFS by using the Montezuma quail reference genome and a minimum base quality of 20 and minimum mapping quality of 20 (Figure S12). Next, we obtained a maximum‐likelihood estimate of the SFS using realSFS by bootstrapping it 100 times and using the mean SFS for each population to estimate per‐site Watterson's theta (*θ*_W_). We estimated heterozygosity for each individual as the total proportion of heterozygous sites from its SFS.

To obtain an estimate of contemporary effective population sizes (*N*_e_) from mean genomic *θ*_W_, we first estimated the whole‐genomic mutation rate (*µ*) for Montezuma quail (*θ*_W_ = 4N_e_ *µ*). Since no linkage map exists for Montezuma quail, we estimated *µ* following Zhan et al. (2013). The Montezuma quail reference assembly was mapped to the chicken genome (*Gallus gallus* GRCg6a) using LASTZ (Harris, 2007). The mean divergence time (*t*) between chicken and Montezuma quail was derived from www.timetree.org , and polymorphic loci were identified only if neither target nor query nucleotide was *N*/*n* and the locus was not in an alignment gap. The final *µ* per nt per year was calculated with the following formula: *µ* = (counts of mutated loci / sequence length) / 2*t* (Zhan et al., 2013).

### 2.7 Genetic differentiation and selection scans

Small populations in isolation can become genetically differentiated due to drift at neutral loci and positive selection at non‐neutral loci (e.g., in response to local adaptation). Both processes lead to nucleotide divergence (*D*_XY_) and divergence in allele frequencies (*F*_ST_) (Matthey‐Doret & Whitlock, 2019; Puzey et al., 2017; Rousset, 1997). We investigated genomic patterns of genetic differentiation by estimating pairwise *F*_ST_ using a sliding window approach (window size=100 kb, step=50 kb) for each population pair (AZ‐TX, TX‐NM, AZ‐NM). We used ANGSD to calculate the 2D SFS for each population pair using the chicken genome (GRCg6a) as reference to polarize alleles as derived or ancestral. We quantified the levels of nucleotide divergence (*D*_XY_) using the calcDxy.R (https://github.com/mfumagalli/ngsPopGen/blob/master/scripts/calcDxy.R). In this case, we ran ANGSD for each population individually to get population‐level AF and GL information, but only for the SNPs previously identified in our genomic dataset. This ensured that sites with a fixed allele in one population were still included in our per population *D*_XY_ calculations.

To identify candidate regions under putative selection due to local adaptation, we Z‐transformed *F*_ST_ around the mean for each sliding window and examined the outliers that had Z(*F*_ST_) values outside 5 standard deviations from the mean (Willoughby et al., 2018). After removing false positives that showed higher deviations due to lack of data (see Results), the remaining outlier windows were inspected for nearby genes. We blasted the 100‐kb outlier window to the chicken genome using default parameters and only retained windows that contained annotated genes with known function.

### 2.8 Population trends and historic demographic sizes

Neutral alleles with rare initial frequencies are more likely to be lost during bottlenecks, whereas more common alleles tend to increase in frequencies more than expected under an equilibrium demographic model. This shift from rarity in the AF spectrum results in an overall positive value of Fu's *F* statistic (Fu, 1997). On the other hand, the addition of de novo mutations in expanding populations tends to produce an excess of rare variants and a negative mean value of Fu's *F*. Fu's *F* is more sensitive to demographic changes than Tajima's D (Ramos‐Onsins & Rozas, 2002) but requires ancestral sequences for unbiased estimations. Thus, we estimated mean Fu's *F* statistic for every population over a sliding window in ANGSD using the chicken genome as an ancestral reference with 100 kb window size and 50 kb step.

We reconstructed ancestral demographic histories using SMC++ v.1.15.2 (Terhorst et al., 2016), which uses unphased whole‐genome data to infer population size histories using sequential Markov coalescent (SMC) simulations. The reads that mapped to the first 10 chicken chromosomes (NC_006088.5–NC_006097.5) comprising ~750 Mbp were used to create composite likelihoods for each population individually by varying the identity of the distinguished individual while keeping other individuals within the population as undistinguished. We used cross‐validation to estimate population size changes using the Powell algorithm with a tolerance of 1 × 10^−5^ and a mutation rate of 3.14 × 10^−09^ (estimated as above). We ran our model using 5000 iterations and used different parameter values for thinning and regularization penalty to avoid degeneracy in the likelihood and overfitting (Terhorst et al., 2016) with final model generated using thinning parameter of 1300 and regularization penalty of 6. A generation time of 1.5 was used to convert generations into years.

### 2.9 Genic diversity and estimation of genetic load

The Montezuma quail genome consists of ~17,500 genes (Mathur et al., 2019), and here, we compared the levels of nucleotide variation across the entire genome to levels of variation in just the genic regions in order to help partition the effects of drift and selection. We used BEDOPS (Neph et al., 2012) to convert the gene annotation file (.gff) to a BED file and filtered BAM files to only include reads that overlapped with the genic coordinates using SAMtools *view*. The GLs and diversity indices were estimated for the genic regions following the same methods and parameters as above. AF at each of the genic variants were calculated from the GL.

Genetic load can be viewed from a gene pool level or at individual level. To distinguish the two perspectives, we introduce the terms potential load and realized load. We quantified the potential load (Load_P_) of a population as the proportion of deleterious variants of different impact classes across all annotated protein‐coding genes. We did so by predicting the effect of each nucleotide variant on the resulting amino acid sequence and then quantifying its putative deleterious impact using SnpEff 4.2 (Cingolani et al., 2014) where we classified only exonic variants that the algorithm considered high quality. The deleterious impact of a variant is predicted under the assumption that the reference allele is nondeleterious, but the alternate allele is deleterious to gene function. A variant is then classified as either high, moderate, low, or no impact based on its inferred effect on protein translation. High‐impact variants should have the most disruptive (i.e., deleterious) effect on protein structures such as premature termination or other loss of function mutations, whereas low‐ or no‐impact mutations were mostly synonymous substitutions with little to no impact on protein sequences. Individuals and populations that bear the highest ratio of highly deleterious mutations to total genic variants have the highest Load_P_. So,

$${\mathrm{Load}}_{P[jk]}=\frac{\mathrm{total}\;\mathrm{number}\;\mathrm{of}\;\mathrm{mutations}\;\mathrm{of}\;\mathrm{impact}\;\mathrm{class}\;i\;\mathrm{in}\;\mathrm{individual}\;j_{k}}{\mathrm{total}\;\mathrm{number}\;\mathrm{of}\;\mathrm{genic}\;\mathrm{mutations}\;\mathrm{in}\;\mathrm{population}\;k}$$

where *i* ∈ (high, moderate, low, no impact) and *k* ∈ (AZ, TX, NM). Load_P_ is conceptually similar to the term “segregating load” (van Oosterhout, 2020), but instead of comparing the absolute number of deleterious SNPs, we defined Load_P_ as a proportion conditioned on all genic SNPs present in a population to standardize load across populations that might vary in levels of genetic diversity or across species that may vary in genome size.

We note, however, that the proportion of potential load that is actually realized in individuals also depends on the mode of dominance and on zygosity. To illustrate how Load_P_ is manifested in terms of individual fitness, we calculated realized load (Load_R_) as the proportion of impactful variants that exist as homozygotes within individual diploid genomes. We computed the per‐individual proportion of deleterious variants of each impact class as the total number of deleterious alleles present within an individual divided by twice the number of segregating sites within each impact class (Simons et al., 2014). Thus,

$${\mathrm{Load}}_{R[jk]}=\frac{\mathrm{total}\;\mathrm{number}\;\mathrm{of}\;\;\mathrm{homozygous}\;\mathrm{mutations}\;\mathrm{of}\;\mathrm{impact}\;\mathrm{class}\;i\;\mathrm{in}\;\mathrm{individual}\;j_{k}}{2\times \;\mathrm{total}\;\mathrm{number}\;\mathrm{of}\;\mathrm{sites}\;\mathrm{of}\;\mathrm{impact}\;\mathrm{class}\;i\;\mathrm{in}\;\mathrm{individual}\;j_{k}}$$

where *i* ∈ (high, moderate, low, no impact) and, in the present study, *k* ∈ (AZ, TX, NM). To assess the zygosity of an impactful mutation in genic regions, we called individual genotypes at SNPs within the genes based on the posterior probability of the genotypes from GL using ANGSD. Genotypes were only called at sites with minimum individual depth of 5× to minimize technical biases (Benjelloun et al., 2019). The inverse relationship between dominance and selection coefficient means that highly deleterious mutations that arise in a population are mostly recessive (Agrawal & Whitlock, 2011). Thus, we demarcate load as “potential” and “realized” to show that the potential genetic load of deleterious recessive alleles that are present in a population may or may not be widely realized within an individual, depending on zygosity (Fu et al., 2014), which varies depending on the degree of inbreeding. Thus, Load_P_ is a population‐level and zygosity‐independent assessment of genetic load, whereas Load_R_ is an individual assessment of genetic load dependent on the homozygosity of deleterious mutations within a given genome. Significant differences in mean load (i.e., Load_P_ and Load_R_ of different impact classes) among different populations were identified using Welch's two‐sample *t*‐test.

## 3 RESULTS

In this study, we collected WGS data from 90 Montezuma quail (AZ = 60, TX = 17, and NM = 13; Figure 1). We generated more than 1.65 billion reads (mean = 18.5 million reads per individual) corresponding to approximately 250 billion bases (mean = 2.8 billion bases per individual; >2× individual coverage). Since these samples were opportunistically collected, we found significant variability in the quality and quantity of DNA sequenced. This stochasticity was evident from sequences generated per individual (Table S1) and their depth and breadth of coverage (Table S1, Figure S1). We removed samples that failed to generate the threshold of 8 million bases (*N* = 10) or were less than 50% of the total reads mapped to the Montezuma quail assembly (*N* = 6). However, we achieved a high level of read mapping for the remainder of the samples (84.4% ±18.1%; Table S1). Ultimately, we analyzed genomic information from 74 individuals (AZ = 52, TX = 15, and NM = 7) that covered 65.1 ± 22.1% (mean ± SD) of the Montezuma quail genome at 2.1 ± 1.3× depth (Table 1).

**TABLE 1:** Summary statistics for sequence coverage, inbreeding coefficients (*F*), per‐site Watterson's theta (*θ*_w_), heterozygosity (*H*), and effective population sizes (*N*_e_) for Montezuma quail populations analyzed in this study

	*N*	Sequence depth (X) (mean ±SD)	Sequence breadth (%) (mean ±SD)	*F* (mean ±SD)	Whole genome		Genic regions		*N*_e_ (95% CI)
					*θ*_W_	*H*	*θ*_W_	*H*	
Arizona	52	2.14 ± 0.78	69.45 ± 14.51	0.05 ± 0.08	5.37 × 10^−4^	0.0014	5.23 × 10^−4^	0.0012	42,795 (42,764–42,825)
Texas	15	1.45 ± 1.82	42.69 ± 30.17	0.33 ± 0.28	4.05 × 10^−4^	0.0009	3.94 × 10^−4^	0.0007	32,208 (32,182–32,234)
New Mexico	7	3.48 ± 1.78	84.16 ± 11.51	0.07 ± 0.08	5.57 × 10^−4^	0.0013	4.47 × 10^−4^	0.0011	36,417 (36,390–36,446)

Note

The diversity indices were calculated for either the whole genome or just the genic regions. Long‐term (evolutionary) *N*_e_ was calculated using an estimated mutation rate of 3.14 × 10^−9^ with 95% CI calculated using standard error in *θ*_w_ estimates. Sequence depth is measured in fold coverage, and breadth is measured as percentage of Montezuma quail assembly mapped by the reads.

Our complete mitogenome analysis detected 39 unique haplotypes in the Arizona population with 239 parsimony‐informative sites shared among them. There were 11 unique Texas haplotypes sharing 171 parsimony‐informative sites, and we found only three unique haplotypes for the New Mexico population with 167 such sites. We found per‐site nucleotide diversity (Π) and Kimura 2‐P pairwise distances to be smaller in the Texas and New Mexico mitogenomes (*p* = 0.03 and *p* = 0.04, respectively) as compared to Arizona. Haplotype diversity (*H*_d_) did not significantly differ between Texas and Arizona mitogenomes (*p* = 0.70) but was significantly smaller in New Mexico as compared to Arizona (*p* = 0.02; Figure S2).

**FIGURE 2:**
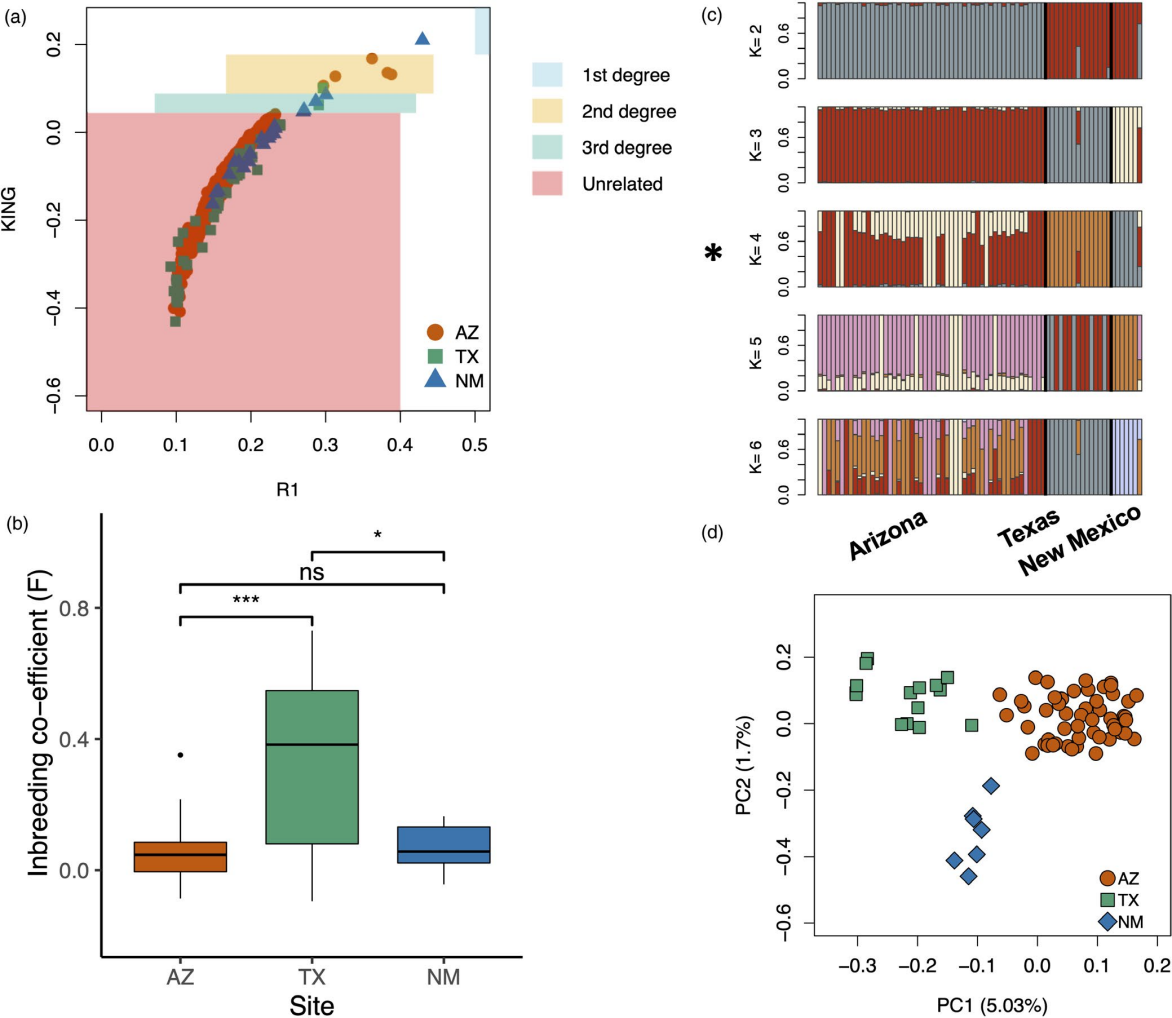
Inbreeding and population structure in Montezuma quail. Samples analyzed in this study were mostly unrelated based on (a) kinship analysis. The degree of kinship (solid boxes) between a pair of individuals was based on Waples et al. (2019). (b) Mean individual inbreeding coefficients (*F*) were significantly higher in the Texas population with no significant difference between Arizona and New Mexico populations. Results from both (c) admixture and (d) PCA clearly demarcate samples from the three collecting sites into independent genetic clusters. However, likelihood estimates indicate the most likely number of ancestral populations in our data is *K* = 4 (indicated with asterisk), where Arizona is sundered into two subpopulations

For the nuclear genome analysis, we partitioned our data into two datasets: population and genomic. The population dataset consisted of GLs from 456,373 SNPs retained from all individuals (*N* = 74). The genomic dataset contained GL information from 6,696,145 SNPs sampled across an equal subset of each representative population (*N* = 21). Using the population dataset, we first estimated the relatedness among our samples to determine whether we had close relatives in the study. Pairwise relatedness was measured for 2341 individual pairs. Almost all the pairs analyzed were either unrelated (99.5%) or 3rd‐degree relatives (0.21%). We found no full‐sibling or parent–offspring relationships (1st‐degree) in our samples; however, five pairs from Arizona, one pair from Texas, and one pair from New Mexico had 2nd degree or half‐sibling relationship (Figure 2a). Overall, our kinship analysis indicates that, consistent with our opportunistic field sampling and broad survey range, close relatives were only rarely sampled and thus should not impact our population structure results. Inbreeding coefficient estimates (Table 1) showed significantly higher levels of mean inbreeding in Texas birds as compared to Arizona birds (Figure 2b; Table S2), whereas inbreeding in Texas was only slightly elevated relative to New Mexico birds. Both PCA and admixture analysis produced similar results indicating that the Arizona, Texas, and New Mexico populations are genetically distinct (Figure 2c,d). However, based on the ∆*K* method (Evanno et al., 2005), the most likely number of ancestral populations is *K* = 4 (Figure S3), splitting Arizona populations into two subpopulations (Figure 2c). The population‐level trends for relatedness, inbreeding, and genetic differentiation were concordant between the two datasets (Figure S4), and thus, it seems clear that sampling issues have not biased our interpretations. This concordance also illustrates that analyzing many SNPs from a small sample can provide similar estimates to analysis from larger sample size, which is often important for endangered species or where sample size is a major restriction.

**FIGURE 3:**
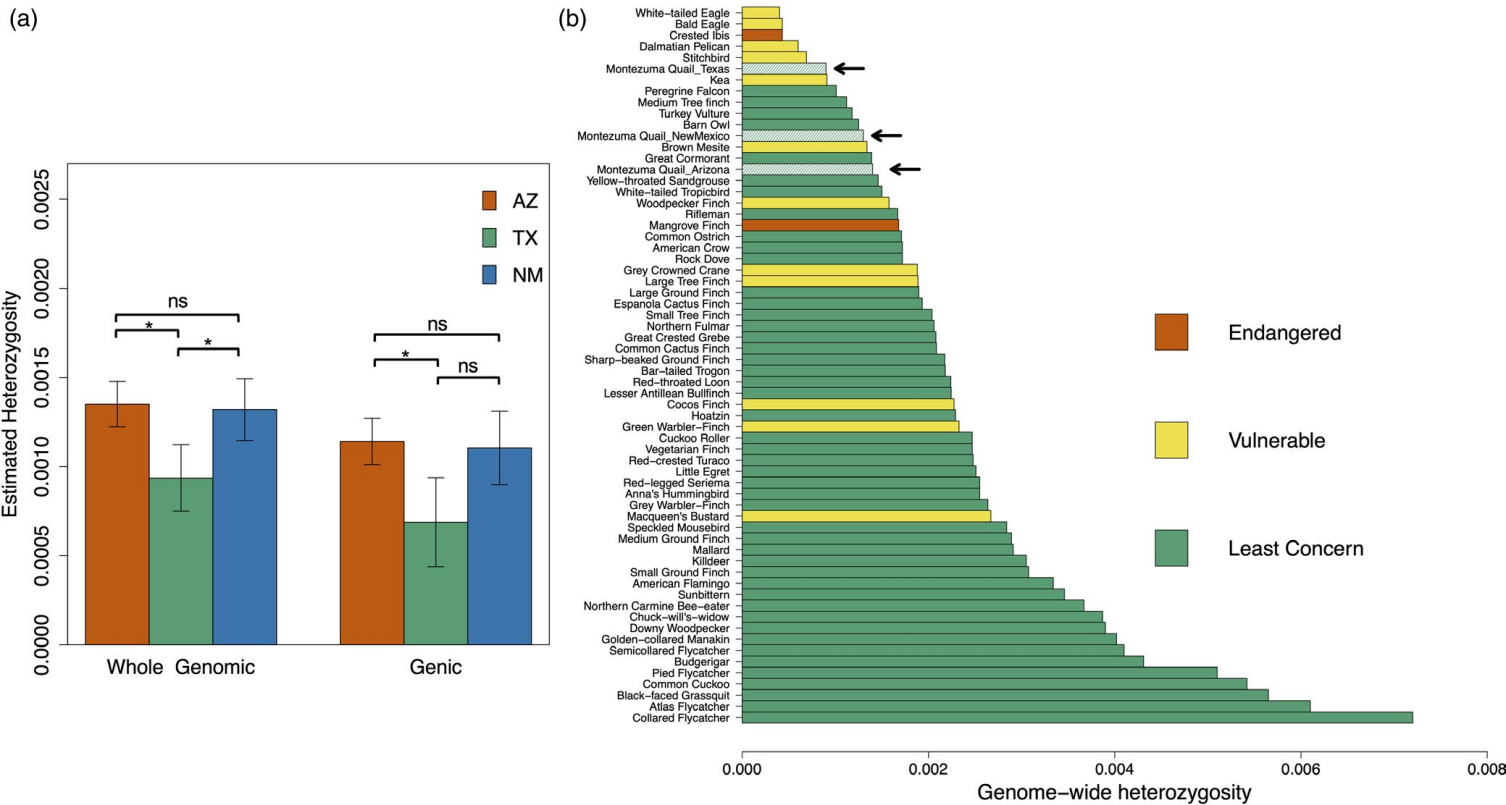
Estimated levels of heterozygosity in Montezuma quail. (a) Genic heterozygosity is comparable to genome‐wide heterozygosity in individuals from all three populations, but Texas quail exhibit significantly lower levels of both as compared to Arizona quail (b) Comparison of genome‐wide heterozygosity with other birds indicates that smaller Montezuma quail populations in Texas and New Mexico have genomic diversity comparable to vulnerable species (Brüniche‐Olsen et al., 2019; de Villemereuil et al., 2019; Li et al., 2014). Heterozygosity was measured as the mean proportion of heterozygous sites per‐individual genome

We used genomic dataset to quantify the levels of genome‐wide nucleotide diversity as estimated by per‐site Watterson's theta (*θ*_W_). Mean genome‐wide *θ*_W_ was significantly lower for the Texas population (*θ*_W_ = 4.05 × 10^−4^; SE = 1.67 × 10^−7^) as compared to both Arizona (*θ*_W_ = 5.37 × 10^−4^; SE = 1.93 × 10^−7^) and New Mexico (*θ*_W_ = 4.57 × 10^−4^; SE = 1.80 × 10^−7^; Table 1; Table S3). The genome‐wide distribution of per scaffold diversity had a higher mean in the Arizona population than in Texas or New Mexico (Figure S5). Contemporary estimates of *N*_e_ were quantified using whole‐genomic *µ* of 3.14 × 10^−9 ^bp^−1 ^year^−1^ (CI: 2.59 × 10^−9^–3.34 × 10^−9^) (Table 1). Thus, Texas quail show a ~30% reduction in their overall genomic diversity with a mean, long‐term evolutionary *N*_e_ reduction of ~25% relative to Arizona. The genomic heterozygosity was also significantly reduced for Texas birds (Table 1) as compared to either Arizona or New Mexico birds (Figure 3a; Table S4). This indicates that smaller Montezuma quail population in Texas is more severely impacted by genetic erosion with contemporary diversity equivalent to those reported in endangered and vulnerable avian species, whereas the larger Arizona population has heterozygosity estimates similar to other more common avian species (Figure 3b).

**FIGURE 4:**
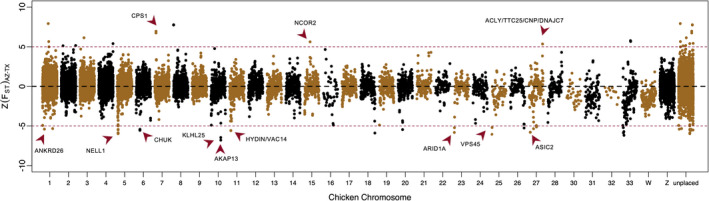
Z‐transformed *F*_ST_ estimates for comparisons between Arizona and Texas samples. The reads were mapped to the chicken genome, and the windows (100 kb width with 50 kb steps) were arranged according to chicken autosomal (1–33) or sex (Z, W) chromosomes. Scaffolds that were not part of the major chicken chromosomes were binned together as unplaced. We found windows within each chromosome that had high (>5 SD) levels of differentiation, and many of those windows contained genes with known function (red arrows). These data illustrate the heterogeneous landscape of genomic differentiation in Montezuma quail

Global estimates of *F*_ST_ between each population pair showed low‐to‐moderate levels of genetic differentiation at the whole‐genome level (Table 2). However, we found significant variation in *F*_ST_ values across the genome for each population pair (Figure 4; Figure S6). One interesting observation was large Z(*F*_ST_) scores for loci on chromosome 16 (NC_006103.5) for all population comparisons (Figure 4; Figure S6). This is probably due to low synteny between quail and chicken at chromosome 16 (Morris et al., 2020), perhaps due to an inversion (Clucas et al., 2019), but this needs further validation using longer sequence scaffolds (Lamichhaney & Andersson, 2019). Low synteny regions had poor mapping quality and thus had missing data that overestimate the differentiation patterns and are marked as outliers with large Z(*F*_ST_) values. Note there is a similar discontinuity at one end of chicken chromosome 26 (Figure S6). We examined the windows that were highly differentiated in both AZ‐TX and TX‐NM comparisons to look for genes and assess their functionality. Genes or a gene clusters associated with the outlier peaks are shown in Figure 4, and their known functions are listed in Table S5. Per‐site *F*_ST_ and *D*_XY_ values for SNPs located in those genes are shown in Fig S7. In total, we found 12 genes that exhibited very high levels of differentiation (>5 SD) with known function in immunity and/or development‐related traits (Table S5). These genes are candidates for those under strong selection and could underlie local adaptations in Texas quail.

**TABLE 2:** Estimates of global *F*_ST_ between the different population pairs measured for either the whole genome or just the genic regions. 95% CI was calculated using standard error in *F*_ST_ estimates by 100 bootstraps of the 2D SFS for each population pair

Population pair	Mean global *F*_ST_ (95% CI)	
	Whole genome	Genic regions
Arizona–Texas	0.1287 (0.1286–0.12878)	0.2042 (0.2018–0.2065)
Texas–New Mexico	0.0962 (0.0961–0.0962)	0.1762 (0.1744–0.1781)
Arizona– New Mexico	0.0972 (0.0972–0.0973)	0.1217 (0.1213–0.1220)

**FIGURE 5:**
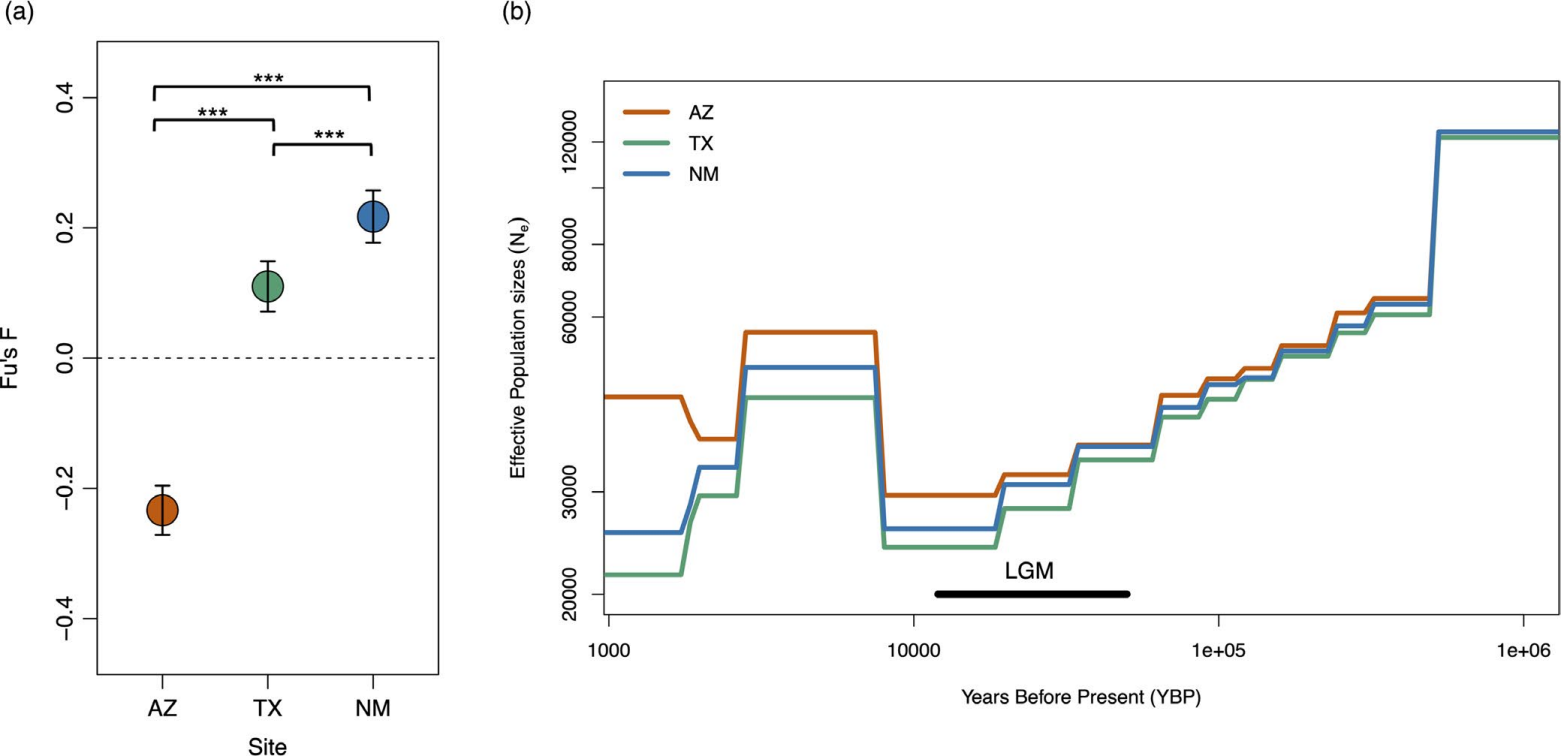
(a) Population trends and (b) demographic histories of Montezuma quail. Population trends indicate that only the Arizona population(s) has been expanding (Fu's *F* < 0), whereas both Texas and New Mexico populations are declining (Fu's *F* > 0). Error bars indicate 95% CI around the estimate. The data indicate that Montezuma quail experienced a strong historic bottleneck during the last glacial maxima (LGM) followed by re‐expansion, and the similar demographic trajectories of each population prior to the LGM suggest that genomic differentiation (Figure 4) is relatively recent in origin

Demographic analysis indicated that the Arizona population have been expanding with Fu's *F* = −0.23 ± 0.01 (mean ± SE), whereas both the Texas and New Mexico populations have been declining with Fu's *F* = 0.11 ± 0.02 and 0.22 ± 0.02, respectively (Figure 5a). We tracked *N*_e_ estimates over the last ~1 million years using the pairwise sequentially Markov coalescent method (Figure 5b). The three populations display concordant trajectories for most of their evolutionary history over that time frame. We observed a decline in *N*_e_ from in the period of 10^6^–10^5^ years before present (YBP) followed by a more stable period. A subsequent re‐expansion occurred around 10,000 years ago, and then, populations began to rebound until growth rates became negative around 3000–5000 YBP (Figure 5b).

One of the major emphases of our study was to assess the adaptive potential of Montezuma quail, particularly in the small, isolated Texas population. Variation in protein‐coding genes has the capacity to accurately gauge adaptive potential (Barbosa et al., 2018). The trend we observed for the subset of genic diversity was similar to the whole‐genome data; in both cases, there was a ~25% reduction in nucleotide diversity in Texas quail (Table 1). In particular, the Texas population had significantly lower (*θ*_W_ = 3.94 × 10^−4^; SE = 2.87 × 10^−7^) genic nucleotide diversity as compared to both Arizona (*θ*_W_ = 5.23 × 10^−4^; SE = 3.33 × 10^−7^) and New Mexico (*θ*_W_ = 4.47 × 10^−4^; SE = 3.09 × 10^−7^; Table S6). Mean heterozygosity (i.e., proportion of heterozygous sites per individual) in the genic regions of Texas quail was significantly reduced relative to Arizona quail, whereas Texas and New Mexico samples showed similar levels of genic heterozygosity (Figure 3a; Table S7). Our *F*_ST_ estimates from the genic regions show significantly higher levels of differentiation among the three populations as compared to the whole‐genomic background (Table 2), which indicates that both selection and drift contribute to population structure (which could also be influenced by recombination and introgression).

**FIGURE 6:**
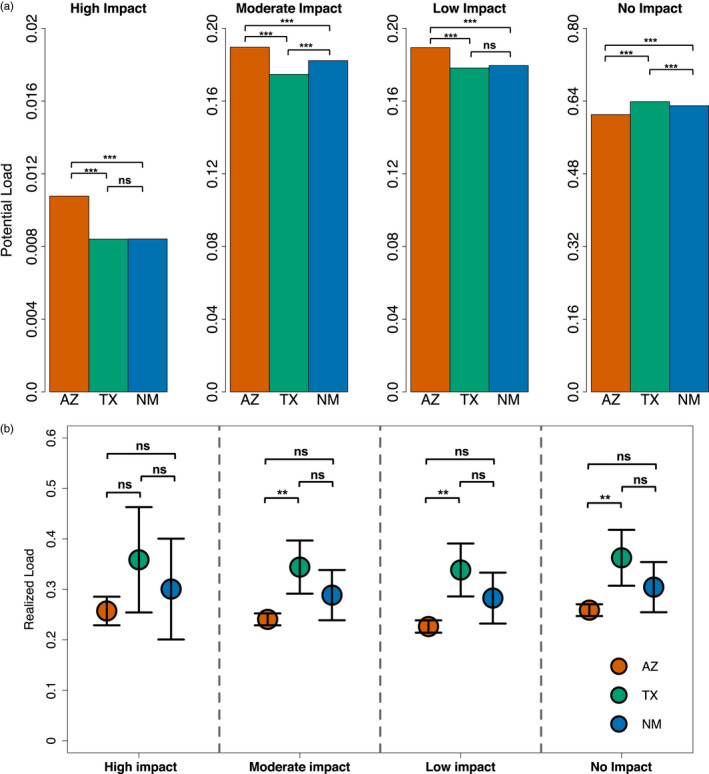
Larger populations have higher potential genetic load, but load is more realized in smaller, inbred populations (see Materials and Methods for details). Variants were classified as either high, moderate, low, or no impact based on their inferred effect on protein translation. High‐impact variants should have the most disruptive (i.e., deleterious) effect, whereas low‐ or no‐impact mutations were mostly synonymous substitutions with little to no impact on protein sequences (a) Potential genetic load was estimated for each population as the proportion of deleterious mutations within annotated protein‐coding genes. The Arizona samples had the highest potential load of high‐impact, moderate‐impact, and low‐impact variants. Note the difference in scales on *y*‐axis. (b) Realized load was measured as the mean frequency of deleterious alleles found within individual genomes for each impact class (*N* = 21; AZ = 7, TX = 7, and NM = 7). No significant difference was found in realized load of highly deleterious mutations between Texas and Arizona quail, but the small Texas population has a higher frequency of moderate‐, low‐, and no‐impact variants coupled with more inbreeding and more homozygosity (Figures 2, 3, and S9) than the larger outbred Arizona populations. Error bars indicate 95% CI around the estimates. ns *p* > 0.05; *p < 0.05; **p < 0.01; ***p < 0.001

To quantify selection and the genetic load associated with the genic variants, we compared the deleterious mutations within protein‐coding genes (Figure S8) and their predicted change on translation (Figure 6a). Most (82.1%) of the genic variation was due to noncoding intronic sites upstream and downstream of the transcription unit; both of these sources of variation can impact gene expression levels and thus serve as sources of regulatory variation. Exonic sites harbored about 4.5% of the genic variation. Within the exonic SNPs, the Arizona population had a significantly higher potential load (Load_P_) due to higher proportions of high‐, moderate‐, and low‐impact deleterious mutations, and lower proportions of noncoding variants, when compared to either the Texas or New Mexico populations (Figure 6a; Table S8). Our estimates of realized load (Load_R_) showed that Texas quail had no significant difference in the mean AF of highly deleterious mutations per individual (Figure S9; *p* > 0.05) as compared to Arizona quail. Most exonic variants were classified as moderate‐ or low‐impact deleterious mutations, and we found them at higher frequencies and significantly more homozygous contributing to significantly higher Load_R_ in Texas quail as compared to Arizona quail (*p* = 0.1 and *p* = 0.3; Figure 6b; Figure S9; Tables S9, S10). We recognize that calling genotypes may be biased due to low coverage (Figure S13) and sample size (Benjelloun et al., 2019), but we note that the observed effects of depth of coverage or mapping rate on our load estimates are statistically insignificant (Tables S11, S12) and the trends we observe here among different impact classes have also been observed in other natural populations (Grossen et al., 2020).

## 4 DISCUSSION

In this study, we analyzed whole‐genome sequences from three natural populations of Montezuma quail that vary in size and habitat continuity (Figure 1) to understand how drivers of genetic erosion (e.g., small sizes and isolation) can affect genomic diversity and reservoirs of future adaptive potential. Small populations are predicted to have lower levels of diversity (Soulé, 1985), and recessive deleterious alleles should have a more pronounced impact on fitness than in large populations due to inbreeding (Charlesworth & Charlesworth, 1999). Populations that have experienced declines and are restricted to smaller habitats tend to have lower levels of overall genomic heterozygosity (Barsh et al., 2017; Brüniche‐Olsen et al., 2019; Palkopoulou et al., 2015), but how these factors affect the adaptive potential is far less explored. By comparing levels of genome‐wide diversity, genic (i.e., potentially adaptive) diversity, and quantifying genetic load in different populations, our aim was to gain a better understanding of how genetic erosion contributes to extinction risks by decreasing the adaptive potential and mean fitness of small populations.

### 4.1 Genetic erosion reduces genomic diversity

Our genomic diversity estimates are consistent with predictions for small declining populations that are expected to be most impacted by genetic erosion (Bijlsma & Loeschcke, 2012; Leroy et al., 2018). Species with small population sizes have lower diversity (Frankham, 1996) and less adaptive potential (Hedrick et al., 2019) than larger populations, and our population genomic data are consistent with these expectations. Montezuma quail exhibit lower levels of whole‐genomic heterozygosity than many other avian species (Figure 3). The reduction in genomic diversity in Montezuma quail is reflective of long‐term declines in *N*_e_ over the last million years (Figure 5b). More specifically, Montezuma quail from Texas are the most genetically depauperate of the populations we surveyed, with genomic diversity similar to vulnerable and endangered birds (Figure 3b). Our Texas samples had genome‐wide heterozygosity similar to raptors and other large birds (Table 1, Figure 3b) even though small birds typically have more genetic diversity (Eo et al., 2011). Overall, we think the data reveal that genomic erosion has likely reduced the evolutionary potential of Montezuma quail in Texas and that this reduction is unlikely to improve without gene flow through assisted translocation or other means.

### 4.2 Isolation leads to more inbreeding

A lack of migration among populations limits gene flow and accelerates inbreeding (Frankham, 1996; Gong et al., 2010; Hedrick et al., 2016; Keller, 2002; Madsen et al., 1996; Pulanić et al., 2008). Our samples from Montezuma quail populations in the United States form independent genetic clusters (Figure 2c,d), which is unsurprising given the geographic distances among sampling sites and the limited dispersal capacity of this ground‐dwelling bird (Stromberg, 1990). These results are in general accordance with our previous findings based on a small SNP panel (Mathur et al., 2019), but the divide in Arizona (Figure 2c; Figure S3) was undetected with that same SNP panel. Our kinship analysis suggests that very few of our samples were derived from related individuals (Figure 2a), and our inbreeding estimates show that the Texas population is highly inbred as compared to Arizona and New Mexico (Figure 2b). Our samples were acquired opportunistically and that likely reduced the probability of collecting related individuals. However, inbreeding itself can reduce estimates of kinship as inbred individuals may have elevated number of alternate homozygous genotypes and a reduced number of shared heterozygous genotypes. We observed an elevated incidence of alternative homozygotes for within‐Texas comparisons (Figure S10), which could lead to longer runs of homozygosity (ROHs). However, we did not explicitly test for differences in ROHs as GL method uses a probabilistic approach to quantify inbreeding coefficients (see Meisner & Albrechtsen, 2018, for details) and is not recommended for ROH analysis. Overall, we think the collective genomic evidence presented herein shows that the small, isolated population of Montezuma quail in West Texas is relatively inbred, meaning more of the potential genetic load will be realized (see below).

### 4.3 Impact of genetic drift on population divergence

One of the major drivers of genetic erosion in small populations is genetic drift. In the absence of migration, genetic drift can fix common alleles or lose rare alleles from the gene pool. Isolated populations with historically low sizes can become phenotypically distinct over time (Holycross & Douglas, 2007; Schierup et al., 2018) due to differences in nucleotide composition (*D*_XY_) (Wakeley, 1996) or allele frequencies (*F*_ST_) (Beaumont, 2005). The intensity of genetic differentiation due to drift is generally expected to be the same for all neutral loci in the nuclear genome due to lack of selection pressures, but it is complicated by linked selection (McVean et al., 2009; Rettelbach et al., 2019). Recent genomic studies have identified “differentiation islands” among populations that could be either due to local adaptation or due to hybridization from an unstudied and genetically differentiated “ghost population” (Burri et al., 2015; Ellegren et al., 2012). We observe similar results in Montezuma quail populations (Figure 4; Figure S6) where many regions show highly significant values of *F*_ST_ even though global estimates seem biologically insignificant (Table 2). Some of these high‐ *F*_ST_ windows no doubt represent statistical artifacts, but many of these highly differentiated regions contain functional genes (Figure 4) that are associated with traits that may be under local selection (Table S5) (Willoughby et al., 2018). These genomic islands of differentiation between populations raise the theoretical possibility that local adaptations could constrain genetic rescue due to the possible reduction in fitness of interpopulation hybrids (Bell et al., 2019; Whiteley et al., 2015). On the other hand, such analyses have the potential to identify source populations that have adaptive genetic signatures most similar to the recipient population and thus the greatest likelihood of success from a long‐term, evolutionary perspective. Furthermore, recent meta‐analyses clearly indicate that the empirical benefits of maximizing overall genetic variation in the target population (e.g., via genetic rescue) clearly outweigh a variety of theoretical risks (Ralls et al., 2020).

### 4.4 The adaptive potential and genetic load of small populations

Understanding the adaptive response of a species to future environmental changes is a high priority for conservation (Holderegger et al., 2019) as this response impacts the long‐term probability of persistence (Hedrick et al., 2019), but such an assessment is not straightforward. Genetic erosion is expected to increase extinction risk by either reducing the overall standing variation thus reducing adaptive potential (Keller, 2002) and/or by decreasing mean fitness due to the accumulation of deleterious mutations (Lynch et al., 1995; Ohta, 1992). We evaluated these two detractors of evolutionary capacity by considering variation contained exclusively in genic regions and assessing their possible phenotypic impact. Montezuma quail have over 17,000 genes, and our results show that both nucleotide diversity and heterozygosity in genic regions are lower relative to the whole‐genomic background (Table 1; Figure 3a). This is not entirely unexpected as many genes might be evolving neutrally or nearly so, but some are highly conserved and mutations arising at these genes will be deleterious and subject to purifying selection (Rettelbach et al., 2019). Our study thus documents a reduction in both the “nearly neutral” (all) and “adaptive” (genic) fractions of genomic diversity in progressively smaller wild quail populations. These reductions in genomic diversity, including both nucleotide diversity and heterozygosity, are likely to diminish the evolutionary potential of the small, isolated Texas population.

The proportion of deleterious mutations present in the genic regions reflects the genetic load of a population (Charlesworth et al., 1993; Ellegren & Sheldon, 2008; Hedrick & Garcia‐Dorado, 2016). We introduced the term potential load (Load_P_) to summarize the population‐wide genetic load dependent on the proportion of deleterious mutations in a given gene pool. Our results indicate that Arizona quail carry significantly more high‐impact deleterious variants as compared to Texas quail, and this difference tends to diminish with variant impact (Figure 6a). This means that overall, larger populations have higher Load_P_ as they harbor more sites that could potentially be deleterious or evade selection. Most of the genic variants are noncoding (Figure S8) and thus do not impact amino acid sequences, but we expect that many serve as regulatory variants that impact expression levels (Harder et al., 2020). Recent population genomic studies have shown via simulations (Coop et al., 2015) and empirical data (Ávila et al., 2010; Do et al., 2015; Rettelbach et al., 2019) that most deleterious genic variants are rare and exist at low frequencies. Over evolutionary timescales, rare deleterious variants tend to be either purged by strong purifying selection or lost due to drift, but at any snapshot in time small‐effect recessive mutations can taint a gene pool (as seen in our Texas quail). Overall, population genomic data are revealing that most populations can efficiently cull highly deleterious mutations, but small‐effect deleterious mutants that escape selection are difficult to purge in small populations where drift predominates (i.e., when *N*_e_(*s*) < 1). In addition to drift, individuals from smaller inbred populations tend to carry these small‐effect deleterious mutants as homozygotes, whereas they tend to be heterozygous in larger outbred populations (Figure S9). To compare the loss of individual fitness due to homogenization of deleterious alleles, we introduced the concept of realized load (Load_R_) to better assess genomic vulnerabilities and estimate inbreeding depression in individuals of small populations. It seems clear that large effect deleterious mutations (e.g., *FOXQ1* (Barsh et al., 2017)) can have a major impact on fitness. However, most adaptive traits are polygenic and based on many small‐effect mutations, so small‐effect deleterious alleles in homozygotes may disproportionately contribute to the overall Load_R_ in small and declining populations like in Montezuma quail from West Texas.

### 4.5 Conservation considerations

Our results indicate that Montezuma quail populations in the United States exhibit low genomic diversity comparable to a number of threatened and endangered species (Brüniche‐Olsen et al., 2019; de Villemereuil et al., 2019; Zhan et al., 2013) (Figure 3b). Our genomic diversity estimates are consistent with predictions for small declining populations, and we argue that our estimates of genic diversity serve as a reasonable proxy for the evolutionary potential of the species. This study adds to the growing body of literature urging conservation organizations such as IUCN to add genetic diversity estimates as a consideration in the listing process (Allendorf et al., 2010; Brüniche‐Olsen et al., 2018; Ralls et al., 2018; Willoughby et al., 2015).

Theory suggests that deleterious mutations should be more abundant in small populations and empirical data support this prediction for species such as wooly mammoths (Barsh et al., 2017) and Iberian lynx (Abascal et al., 2016), with critically low population sizes and ineffective purifying selection. However, most of the species that are declining due to recent anthropogenic activities (like Montezuma quail) have maintained relatively large *N*_e_ with previous cycles of bottlenecks and re‐expansions ((Nadachowska‐Brzyska et al., 2015); Figure 5b). This study and a recent overview of mammals (van der Valk et al., 2019) suggest that smaller populations have significantly lower proportions of deleterious mutations as compared to larger, more genetically diverse populations. These deleterious variants are maintained at lower frequencies and presumably represent a major fraction of the potential genetic load. This pattern exists in part because purifying selection against partially recessive deleterious recessive alleles is relaxed in large populations where higher heterozygosity effectively hides these alleles from selection. In contrast, small populations are only likely to purge strongly deleterious mutations, but the collective genetic load of mildly deleterious mutations still impacts individual fitness when these variants become homozygous due to inbreeding and/or drift. Thus, our genomic data illustrate and quantify the incidence of potential genetic load in large populations (Arizona) relative to the realized genetic load in small, inbred populations such as Texas.

## 5 CONCLUSIONS

We analyzed whole‐genome sequences from different populations of Montezuma quail in the United States and compared the relative impact of genetic erosion between populations of various sizes. Our results indicate that Montezuma quail populations in the United States have mean genome‐wide heterozygosity comparable to other avian taxa of conservation concern. We found that inbreeding and random drift due to isolation are likely the major driving force behind these observed patterns of reduced genomic diversity. We also identified highly differentiated candidate genes that may underlie local adaptations, though we acknowledge a lack of environmental data supporting this idea. More interestingly, we find that larger populations carry a larger proportion of deleterious mutations (potential genetic load) than small populations. However, small populations are most susceptible to reduced fitness because small‐effect deleterious alleles are homogenized due to drift and inbreeding (realized genetic load). Overall, we think these data will be useful to those interested in the conservation of Montezuma quail and that they illustrate the power of population genomics in evaluating adaptive potential in light of fragmented landscapes and rapid environmental change.

## CONFLICT OF INTEREST

The authors declare no conflict of interest.

## Supporting information

Supplementary Material

Table S1

## Data Availability

The sequence datasets generated during the current study are available in NCBI’s Short Read Archive BioProject Accession No. PRJNA623948, BioSample Accession No. SAMN14562436‐509, and SRA Accession No. SRR11514056‐129. The scripts developed for analysis can be publicly accessed at https://github.com/samarth8392/MQU_PopGenomics.
