# Supplementary material for: Genetic load has potential in large populations but is realized in small inbred populations
Source: Evol Appl. 2021 Apr 10;14(6):1540–57. doi: 10.1111/eva.13216 (PMC8210801; doi:10.1111/eva.13216)

Table S1: Whole genome sequence read statistics for each individual (N=74) provided as a separate excel file (Table S1.xlsx)

Table S2: Test for significant differences in mean individual inbreeding coefficients (F) using Wilcoxon rank sum test with continuity correction

| Population Comparison | Test Statistic (W) | p-value |
| --- | --- | --- |
| AZ- TX | 149 | <0.0003 |
| TX-NM | 82 | 0.039 |
| AZ-NM | 149 | 0.446 |

Table S3: Test for significant differences in mean whole-genomic nucleotide diversity estimates (θ_w_) using Wilcoxon rank sum test with continuity correction

| Population Comparison | Test Statistic (W) | p-value |
| --- | --- | --- |
| AZ- TX | 185570578 | < 2.2e-16 |
| TX-NM | 99585686 | < 2.2e-16 |
| AZ-NM | 174571874 | < 2.2e-16 |

Table S4: Test for significant differences in mean individual genome-wide heterozygosity using Wilcoxon rank sum test with continuity correction

| Population Comparison | Test Statistic (W) | p-value |
| --- | --- | --- |
| AZ- TX | 42 | 0.02622 |
| TX-NM | 8 | 0.03788 |
| AZ-NM | 33 | 0.3176 |

Table S5: ﻿ Genes annotated within highly differentiated outlier regions with Z(F_ST_) > 5SD and their chromosomal location in the chicken genome

| **Gene title** | **Gene name** | **Chr** | **Position (bp)** | **Functional description** |
| --- | --- | --- | --- | --- |
| ANKRD26 | Ankyrin repeat domain 26 | 1 | 24435899 - 24484038 | Interacts with proteins or protein complexes |
| NELL1 | Neural EGFL like 1 | 5 | 2461252 - 2747574 | Involved in cell growth regulation and differentiation |
| CHUK | Component of inhibitor of nuclear factor kappa B kinase complex | 6 | 9980703 -10003570 | Has a role in NF-kappa-B signaling pathway which is activated in response to cellular stresses |
| CPS1 | Carbamoyl-phosphate synthase 1 | 7 | 2842238 - 2944989 | Involved in amino acid and nitrogen metabolism |
| KLHL25 | Kelch like family member 25 | 10 | 14396164 - 14410877 | Related role in innate immune system for antigen processing and presentation |
| AKAP13 | A-Kinase Anchoring Protein 13 | 10 | 14421126 -14487012 | Functions as scaffolding proteins to coordinate a Rho signaling pathway |
| HYDIN | Axonemal central pair apparatus protein | 11 | 1545049 - 1649225 | Required for ciliary motility and neural cell development |
| VAC14 | Component of PIKFYVE Complex | 11 | 1649800 - 1700757 | Encodes a scaffold protein for components of cellular membranes |
| NCOR2 | Nuclear receptor corepressor 2 | 15 | 4857712 - 5022472 | Mediates transcriptional silencing of certain target genes |
| ARID1A | AT-rich interaction domain 1A | 23 | 142792 - 202595 | Involved in transcriptional activation and repression of select genes |
| VPS45 | Vacuolar protein sorting 45 homolog | 25 | 83517 - 105457 | Involved in vesicle transport to vacuoles suggesting a role in protein trafficking |
| ASIC2 | Acid sensing ion channel subunit 2 | 27 | 4186435 - 4599845 | Encodes sodium channels that play a role in neurotransmission |
| ACLY | ATP citrate lyase | 27 | 7520780 - 7549243 | Primary enzyme responsible for fatty acid synthesis and carbohydrate metabolism |
| TTC25 | Tetratricopeptide repeat domain 25 | 27 | 7545735 - 7553230 | Localizes to ciliary axonmenes and required for cell signaling and cell motility |
| CNP | 2',3'-cyclic nucleotide 3' phosphodiesterase | 27 | 7553422 - 7558601 | Expressed exclusively by oligodendrocytes in the Central Nervous System |
| DNAJC7 | DnaJ heat shock protein family (Hsp40) member C7 | 27 | 7558532 - 7577845 | Involved in cellular response to heat stress |

Table S6: Test for significant differences in mean genic nucleotide diversity estimates (θ_w_) using Welch Two Sample t-test

| Population Comparison | Test Statistic (t) | Degree of Freedom (df) | p-value |
| --- | --- | --- | --- |
| AZ- TX | 293.8 | 605670000 | < 2.2e-16 |
| TX-NM | -124.58 | 614770000 | < 2.2e-16 |
| AZ-NM | 168.5 | 615040000 | < 2.2e-16 |

Table S7: Test for significant differences in mean individual genic heterozygosity using Wilcoxon rank sum test with continuity correction

| Population Comparison | Test Statistic (W) | p-value |
| --- | --- | --- |
| AZ- TX | 42 | 0.02622 |
| TX-NM | 9 | 0.05303 |
| AZ-NM | 32 | 0.3829 |

Table S8: Test for significant differences in mean proportion of different impact deleterious mutations (potential load) and non-coding variants using Welch Two Sample t-test

| Variant Impact | Population Comparison | Test Statistic (t) | Degree of Freedom (df) | p-value |
| --- | --- | --- | --- | --- |
| High Impact (Most deleterious) | AZ- TX | -3.8785 | 1483.1 | 0.0001097 |
|  | TX-NM | 0.46383 | 1853.6 | 0.6428 |
|  | AZ-NM | -3.2795 | 1974.8 | 0.001058 |
| Moderate Impact (Mildly deleterious) | AZ- TX | -1.5576 | 36808 | 0.004807 |
|  | TX-NM | -1.7429 | 38923 | 0.008135 |
|  | AZ-NM | -3.4915 | 49694 | 0.00193 |
| Low Impact (Least deleterious) | AZ- TX | 11.154 | 38746 | < 2.2e-16 |
|  | TX-NM | -16.953 | 39738 | 0.1193 |
|  | AZ-NM | -7.4833 | 48806 | < 2.2e-16 |
| No Impact  (Non-deleterious) | AZ- TX | 37.876 | 155430 | 2.2e-16 |
|  | TX-NM | -39.807 | 142788 | 2.315e-06 |
|  | AZ-NM | -4.7239 | 175817 | 2.2e-16 |

Table S9: Test for significant differences in mean deleterious allele frequency per individual (realized load) of different impact deleterious mutations and non-coding variants using Welch Two Sample t-test

| Variant Impact | Population Comparison | Test Statistic (t) | Degree of Freedom (df) | p-value |
| --- | --- | --- | --- | --- |
| High Impact (Most deleterious) | AZ- TX | -1.8376 | 6.8798 | 0.1095 |
|  | TX-NM | 0.78681 | 11.977 | 0.4467 |
|  | AZ-NM | -0.81904 | 6.9591 | 0.4399 |
| Moderate Impact (Mildly deleterious) | AZ- TX | -1.9195 | 6.6042 | 0.0098 |
|  | TX-NM | 0.7666 | 11.962 | 0.4582 |
|  | AZ-NM | -0.93947 | 6.676 | 0.3802 |
| Low Impact (Least deleterious) | AZ- TX | -2.0804 | 6.6495 | 0.0182 |
|  | TX-NM | 0.76448 | 11.982 | 0.4594 |
|  | AZ-NM | -1.0863 | 6.7014 | 0.3149 |
| No Impact (Non-deleterious) | AZ- TX | -1.8327 | 6.5348 | 0.0112 |
|  | TX-NM | 0.7811 | 11.863 | 0.45 |
|  | AZ-NM | -0.89278 | 6.6627 | 0.4031 |

Table S10: Test for significant differences in mean minor allele frequency of different impact deleterious mutations variants using Welch Two Sample t-test

| Variant Impact | Population Comparison | Test Statistic (t) | Degree of Freedom (df) | p-value |
| --- | --- | --- | --- | --- |
| High Impact (Most deleterious) | AZ- TX | -1.8785 | 1483.1 | 0.3102 |
|  | TX-NM | 0.46383 | 1853.6 | 0.6428 |
|  | AZ-NM | -1.2795 | 1974.8 | 0.1058 |
| Moderate Impact (Mildly deleterious) | AZ- TX | -1.5576 | 36808 | 0.0048 |
|  | TX-NM | -1.7429 | 38923 | 0.1193 |
|  | AZ-NM | -3.4915 | 49694 | 0.08135 |
| Low Impact (Least deleterious) | AZ- TX | 11.154 | 38746 | < 2.2e-16 |
|  | TX-NM | -16.953 | 39738 | < 2.2e-16 |
|  | AZ-NM | -7.4883 | 48806 | 7.093e-14 |
| No Impact (Non-deleterious) | AZ- TX | 37.876 | 155430 | < 2.2e-16 |
|  | TX-NM | -39.807 | 142788 | < 2.2e-16 |
|  | AZ-NM | -4.72391 | 175817 | 2.315e-06 |

Table S11: Test for significance in correlation between mapping statistics (depth of coverage and percentage of mapped reads) and realized load per individual in each population using linear regression. Load seems to be negatively correlated with the depth of coverage (negative slope) and positively correlated with mapping rate (positive slope). However, the only statistically significant relationship was between depth and load for Arizona individuals (highlighted in bold). The lack of statistical significance shows that our genomic dataset is robust even for samples with low coverage of sequences (see Fig.S13)

| Mapping statistic | Population | Intercept (a) | Slope (b) | R^2^ | p-value |
| --- | --- | --- | --- | --- | --- |
| Depth of coverage | **AZ** | **0.63853** | **-0.10642** | **0.6812** | **0.02222** |
|  | TX | 0.403528 | -0.003754 | 0.02471 | 0.7364 |
|  | NM | 0.36789 | -0.01318 | 0.05276 | 0.6203 |
| Mapping rate (%) | AZ | -0.215863 | 0.004967 | 0.08275 | 0.5316 |
|  | TX | 0.245507 | 0.001754 | 0.1193 | 0.4479 |
|  | NM | -6.54206 | 0.07058 | 0.1376 | 0.4126 |

Table S12: Test for significance in correlation between depth of coverage and realized load per individual in each impact class using linear regression. Load seems to be negatively correlated with the depth of coverage (negative slope). However, none of these correlations were statistically significant. The lack of statistical significance shows that our genomic dataset is robust even for samples with low coverage of sequences (see Fig.S13)

| Variant Impact | Intercept (a) | Slope (b) | R^2^ | p-value |
| --- | --- | --- | --- | --- |
| High Impact (Most deleterious) | 0.38180 | -0.01805 | 0.06621 | 0.1364 |
| Moderate Impact (Mildly deleterious) | 0.37155 | -0.01931 | 0.08185 | 0.1117 |
| Low Impact (Least deleterious) | 0.35595 | -0.01564 | 0.03852 | 0.1954 |
| No Impact (Non-deleterious) | 0.40170 | -0.02317 | 0.1354 | 0.05628 |

Figure S1: Individual depth of coverage (X) and breadth of coverage (%). Depth of coverage is calculated as the mean base coverage covered by the reads and breadth is calculated as the percentage of draft Montezuma quail covered by the reads.


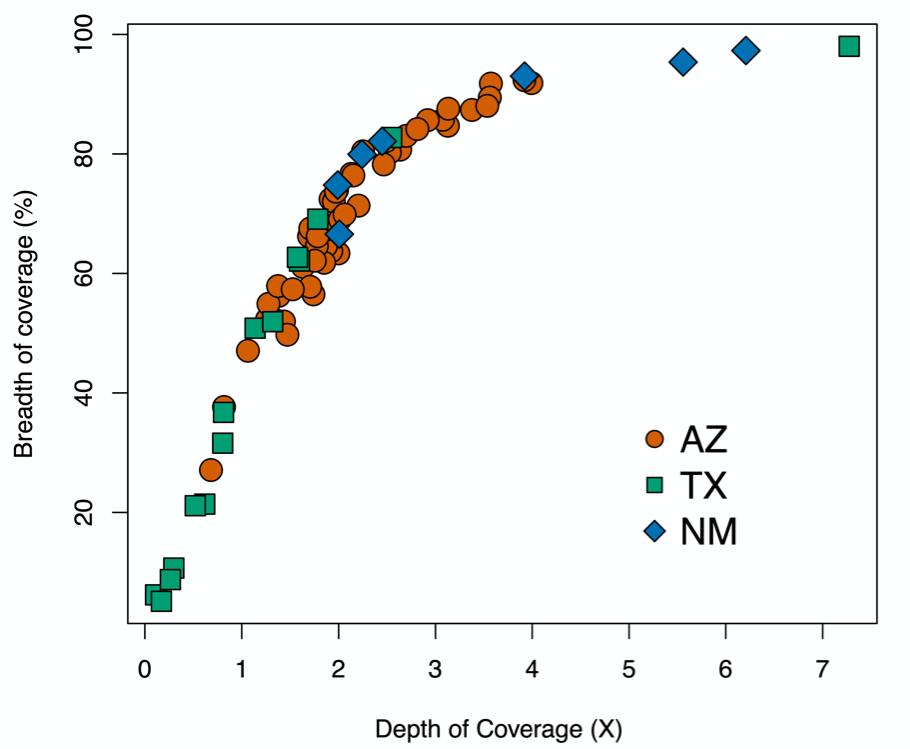


Figure S2: ﻿Mitogenome diversity statistics for each population. (A) Per-site nucleotide (Π), (B) Within population genetic distances calculated using Kimura-2P parameter (K-2P), and (C) Haplotype diversity in Montezuma quail populations in Arizona, Texas, New Mexico. Π and Kimura 2-P pairwise distances were smaller in the Texas and New Mexico mitogenomes (p=0.034 and p=0.041 respectively) as compared to Arizona but no significant difference in haplotype diversity between Texas and Arizona mitogenomes (H_d_ ; p = 0.7) but significantly smaller in New Mexico (p=0.02).


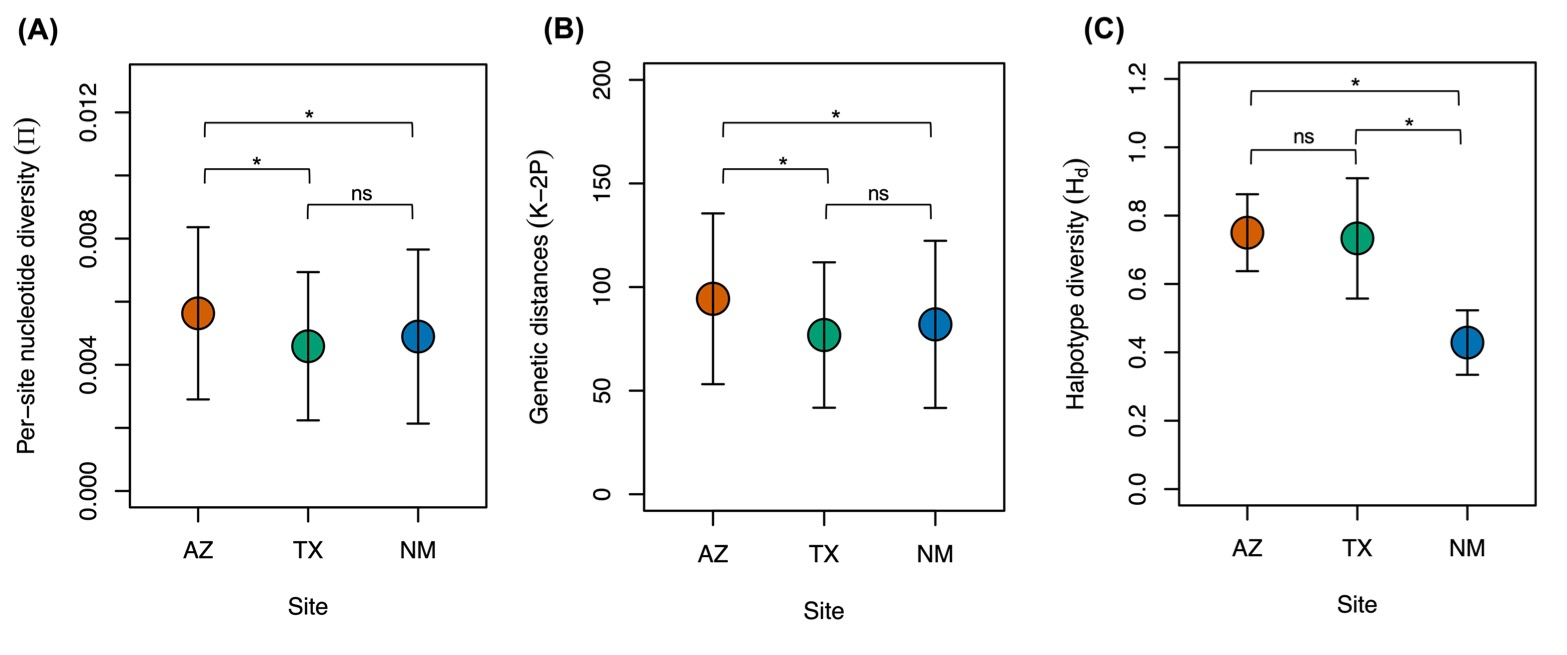


Figure S3: Delta K values (for each K = 1-10) indicate the best estimated number of ancestral populations is K = 4 based on Evanno et al. (2005). At K=4, the Arizona, Texas, and New Mexico populations form independent clusters with Arizona populations being split into two subpopulations.


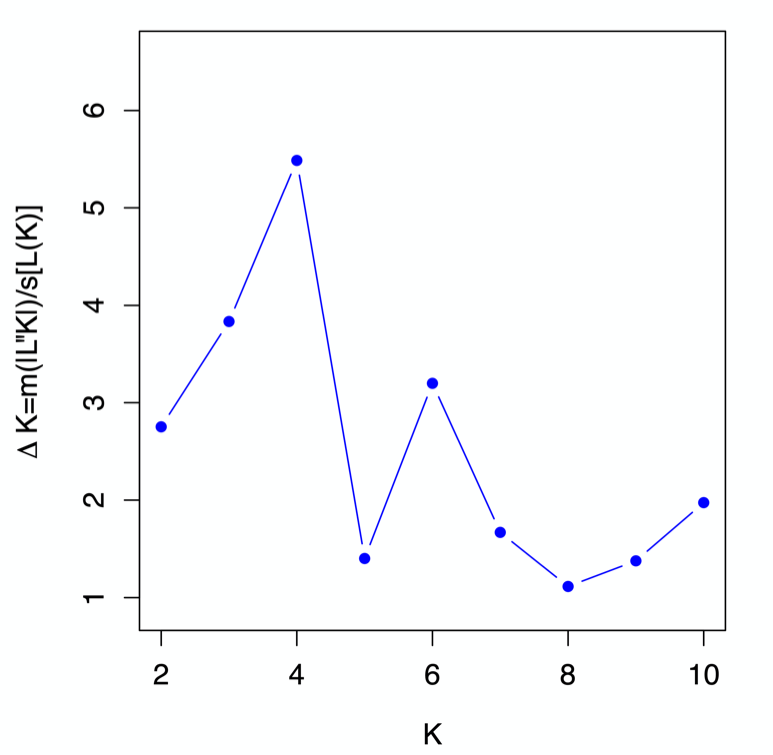


Figure S4: (A) Pairwise Relatedness comparisons, (B) Inbreeding coefficient estimation, (C) PCA, and (D) Admixture, estimates *using equal subsamples* (N=21; AZ=7, TX=7, NM=7). These results are similar to what was estimated using population dataset (N=74; AZ=52, TX=15, NM=7) indicating that the results shown in the main text are biologically relevant and not due to our sampling scheme.

Figure S5: Distribution of Watterson’s theta (θ_W_) across the whole genome. θ_W_ was calculated for every window (100kb size, 50kb step)

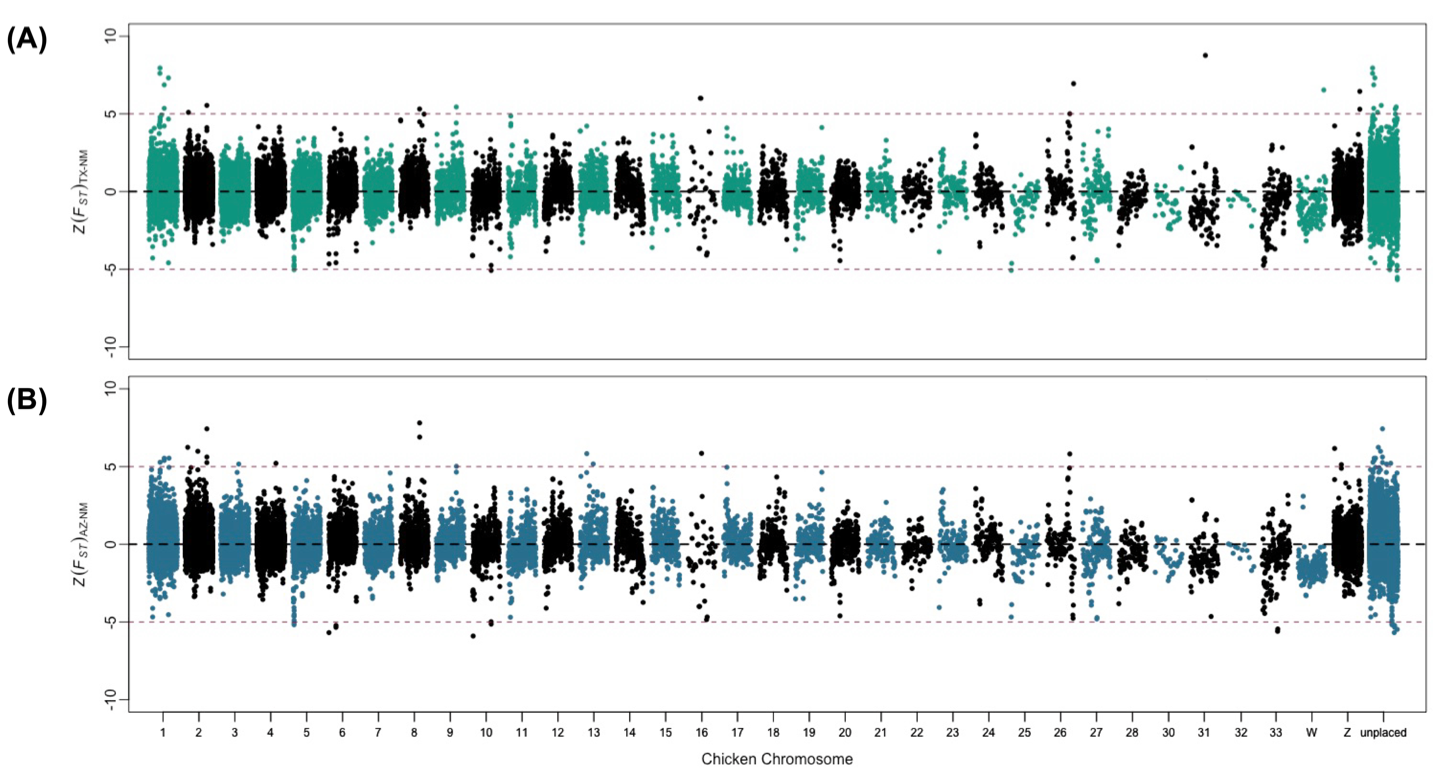
Figure S6: Z-transformed F_ST_ estimates for comparisons made between (A) Texas and New Mexico Montezuma quail, and (B) Arizona and New Mexico Montezuma quail populations for every 100 kb window (50 kb steps). The reads were mapped to the chicken genome and the windows were arranged according to chicken autosomal (1-33) or sex (Z, W) chromosomes. Scaffolds that were not part of the major chicken chromosomes were binned together as unplaced. This figure shows a heterogeneous landscape of genetic differentiation and drift is the primary evolutionary driver behind the observed patterns.


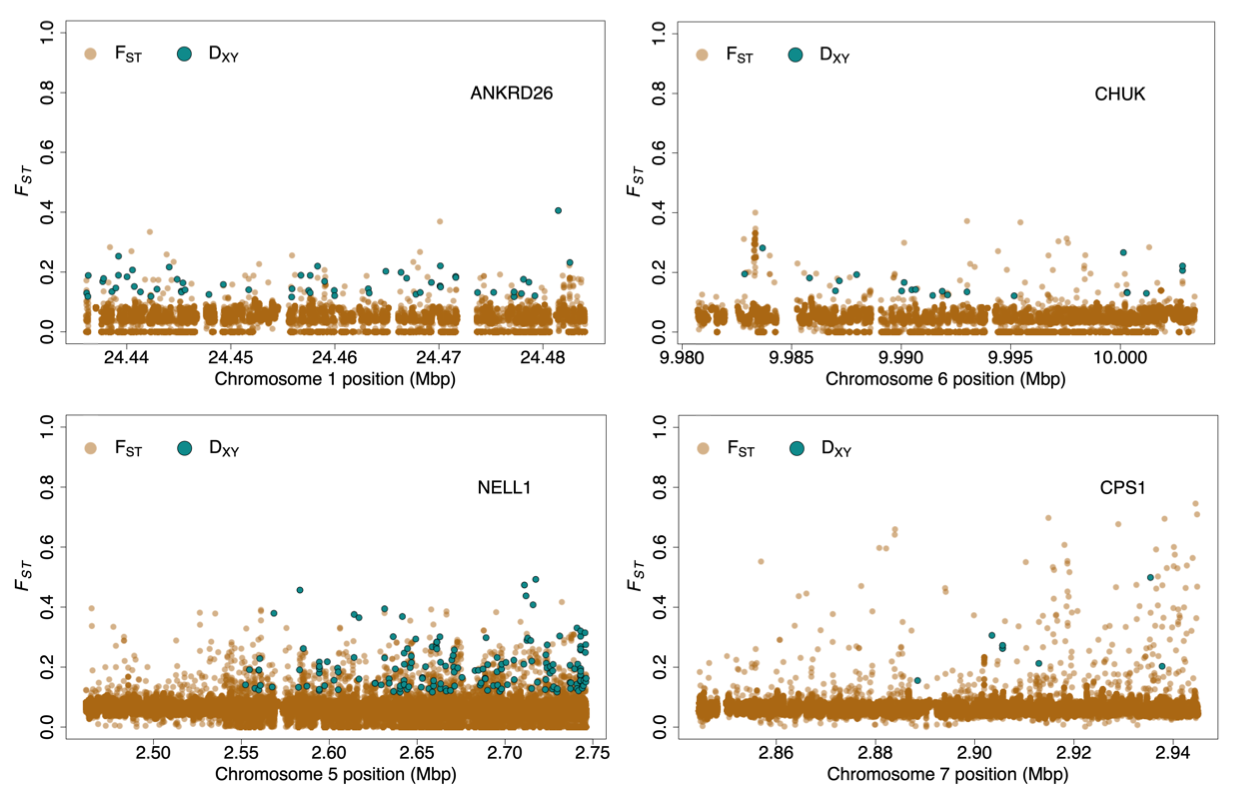
Figure S7: ﻿Pairwise F_ST_ for each polymorphic site in the genes associated with outlier windows in Arizona and Texas populations and D_XY_ for the SNPs that were segregating in all three populations. The functional description of each gene is listed in Table S5.


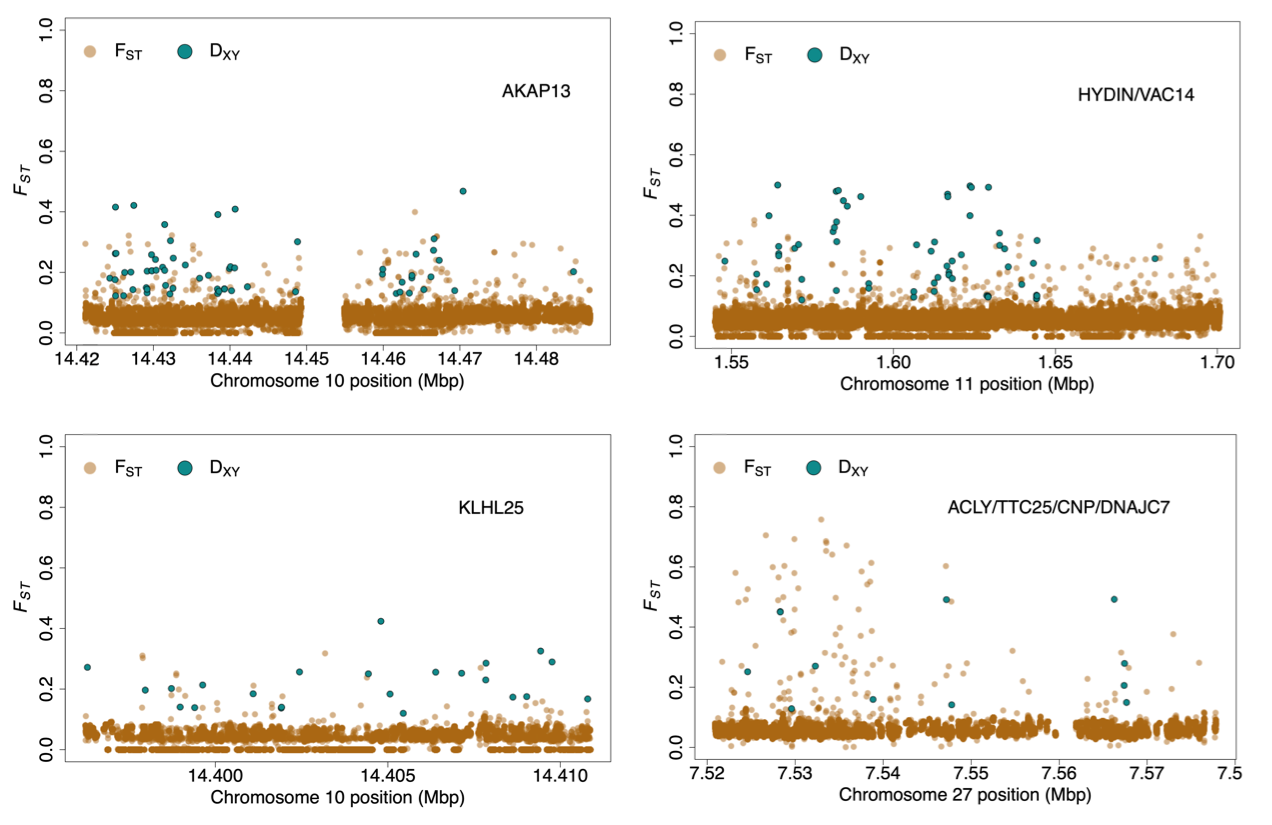
Figure S7: Continued…


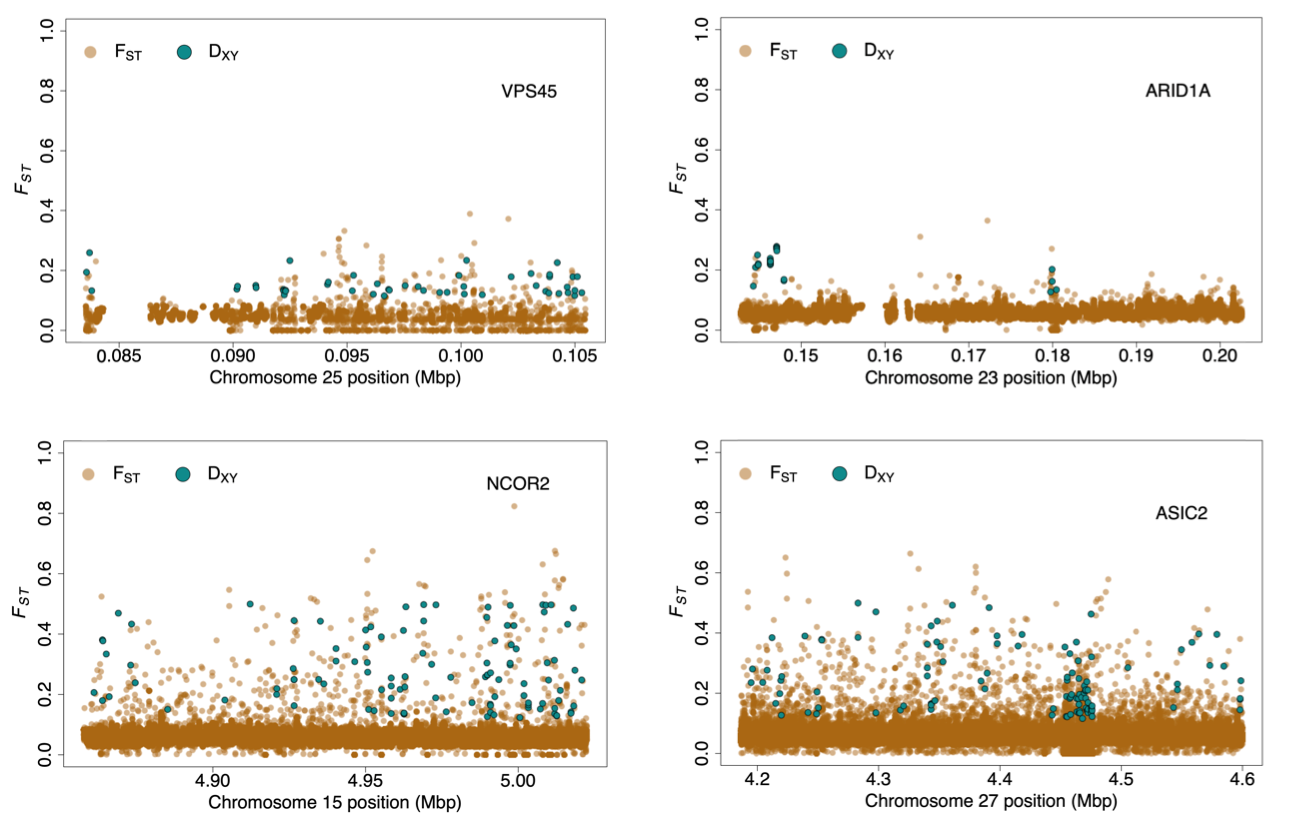
Figure S7: Continued…


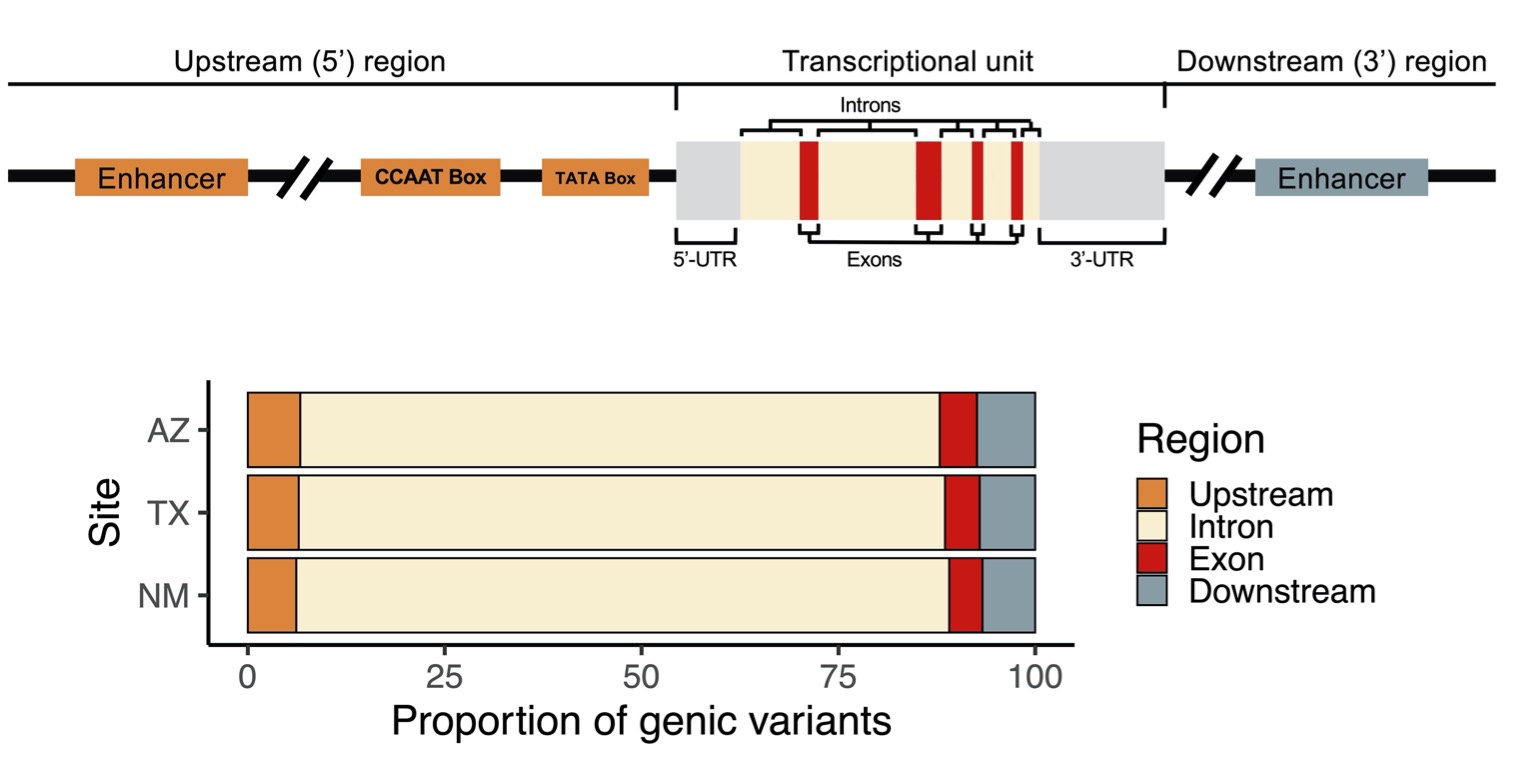
Figure S8: Schematic of eukaryotic gene structure and proportion of variants in different genic regions where the colors in each panel correspond to one another. Much of the genic variation exists outside the transcription unit.

Figure S9: Mean minor allele frequency for different impact class variants. Error bars represent 95% CI.

Figure S10: Within population comparisons for R0 statistics from kinship analysis. This statistic is estimated from ﻿genome-wide pattern of two individuals sharing identity by state. R0 is the ratio of alternate homozygous genotype likelihoods to shared heterozygotes observed at each polymorphic site between a comparison of two individuals. A mean higher than 1 indicates that the two individuals have more alternate homozygote alleles observed across the genome than shared heterozygote genotypes. Inbreeding between individuals elevates the number of alternate homozygous genotypes and reduces number of shared heterozygous genotypes and was found to be in case of Texas comparisons. Error bars indicate 95% CI around mean.

Figure S11: Root mean square error (RMSE) minimization for inbreeding estimation. One issue with iterative algorithms is the stopping criteria. Since we had low coverage WGS data, we used low thresholds (1e-9) and high number of iterations (5000) as stopping criteria of maximization (EM) algorithm for estimating population allele frequencies as well as per-site and per-individual estimates of inbreeding co-efficient. This ensured that the estimation did not stop at the local minima and the estimates were quantified only after the RMSE values were at their global minimum.

Figure S12: Folded site-frequency spectrum (SFS) of different Montezuma quail populations. We only used the genomic dataset (N=7 for each population) to avoid biases SFS due to uneven sample sizes and heavy data pruning. We used ANGSD to generate a folded SFS by using the Montezuma Quail reference and bootstrapped it 100 times. Barplot shows the mean and error bars represent 1 standard deviation (SD) from the mean. The figure represents the proportion of polymorphic sites within the population based on sampling k-derived alleles. Lower allele count represent rarer mutations vs higher alleles counts represent most common mutations. Arizona (AZ) population has higher proportions of rarer mutations as compared to Texas (TX) or New Mexico (NM) populations, which is expected in larger increasing populations. Whereas, TX population has been declining which removed rarer mutations while maintaining intermediate mutations within the genome.

Figure S13: Relationship between mapping statistics and estimated individual load of deleterious mutations of impact class high, moderate, low, and no-impact. (A) Realized load was negatively correlated with individual depth of coverage; however, the relationship was only statistically significant in Arizona (AZ) but non-significant in Texas (TX) or New Mexico (NM) samples (Table S11). (B) Realized load was positively correlated with percentage of reads mapped to the reference genome (Mapping rate) but the relationship was not statistically significant for any population (Table S11). (C) When all samples are taken together, the only significant correlation between load and depth was for no-impact mutations (Table S12). This indicates that our genetic load estimates are robust to the variation in sequence quality of our genomic dataset (N=21; AZ=7, TX=7, NM=7)


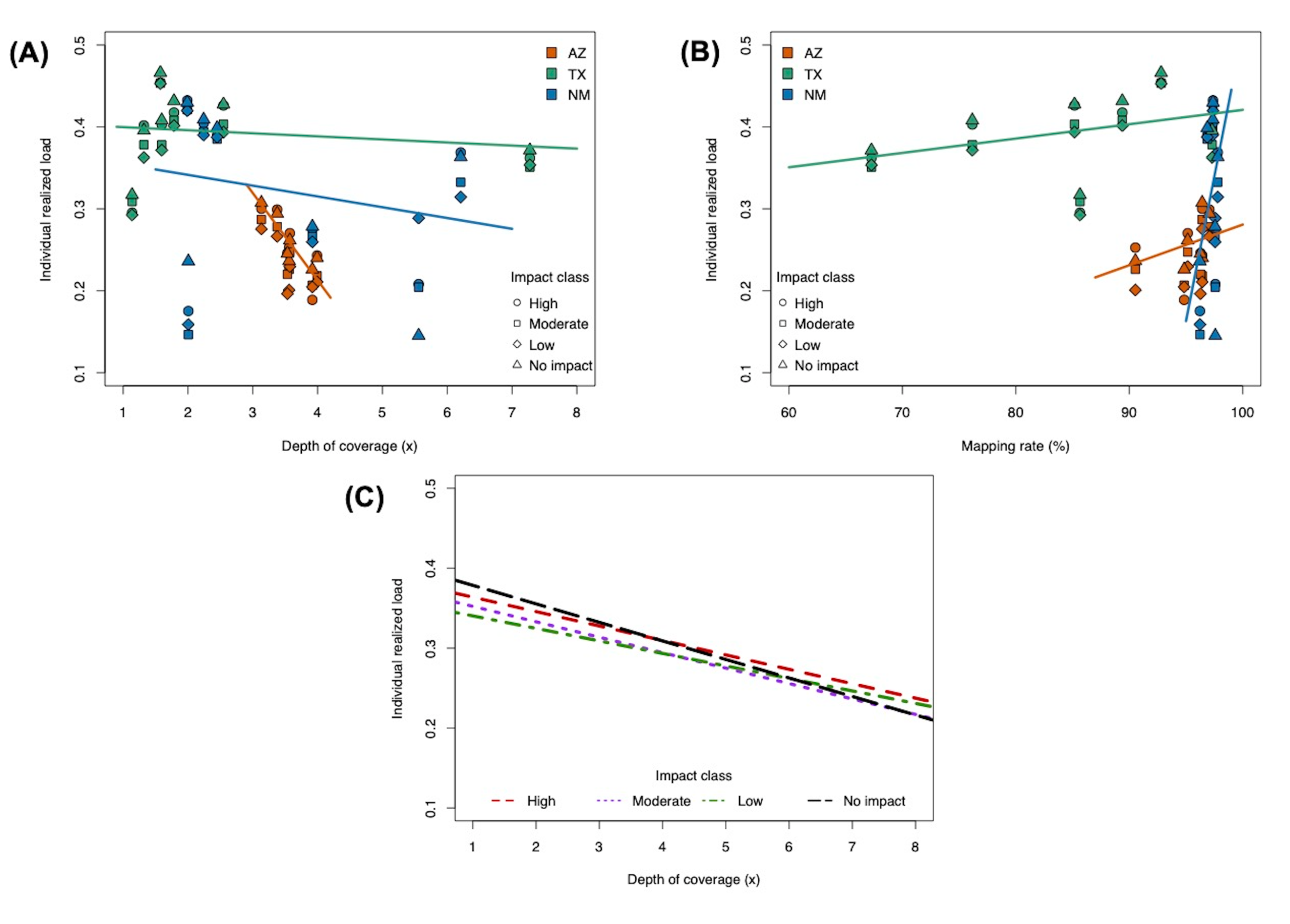

Supplement: Supplementary file 1 — Supplementary Material [file EVA-14-1540-s001.docx]
